# Tracking Interphase Growth at Alloy Anode Interfaces in Sulfide Solid-State Batteries

**DOI:** 10.1021/jacs.5c15251

**Published:** 2025-12-31

**Authors:** Won Joon Jeong, Douglas Lars Nelson, Congcheng Wang, Sun Geun Yoon, Donghyeok Roh, Elif Pınar Alsaç, Kelsey Anne Cavallaro, Lincoln Crowe, Matthew T. McDowell

**Affiliations:** † School of Materials Science and Engineering, 1372 Georgia Institute of Technology, Atlanta, Georgia 30332, United States; ‡ George W. Woodruff School of Mechanical Engineering, Georgia Institute of Technology, Atlanta, Georgia 30332, United States; § School of Chemical and Biomolecular Engineering, Georgia Institute of Technology, Atlanta, Georgia 30332, United States

## Abstract

The chemical stability of solid-state electrolytes (SSEs) in contact with negative electrode materials is essential to enable high performance and safety of solid-state batteries (SSBs). While interphase layers are known to form between Li metal and various sulfide SSEs, there is a lack of understanding of interphase growth in contact with other promising anode materials, such as silicon and aluminum alloys. Here, we track and quantify interphase growth rate, thickness, and composition of various alloy anode thin films in contact with the widely used argyrodite Li_6_PS_5_Cl SSE. Using coulometric titration time analysis (CTTA), we find that the average interphase thickness on four alloy anode materials (Ag, Al, Si, and Ge) is less than half that of pure Li metal after 400 h of growth. Furthermore, the interphase growth rate is strongly dependent on the applied stack pressure and varies among the different alloy materials. The interfacial contact area, which is governed by alloy mechanical properties and deformation under stack pressure, is found to be a critical factor in determining interphase growth rate. Time-of-flight secondary-ion mass spectrometry further confirmed thinner and uniform interphase growth on alloy anodes compared to Li metal. This study bolsters our understanding of interfacial stability of various alloy anode materials married with Li_6_PS_5_Cl SSE, and it suggests that alloy anodes could exhibit enhanced stability compared to Li in sulfide SSB applications.

## Graphical abstract

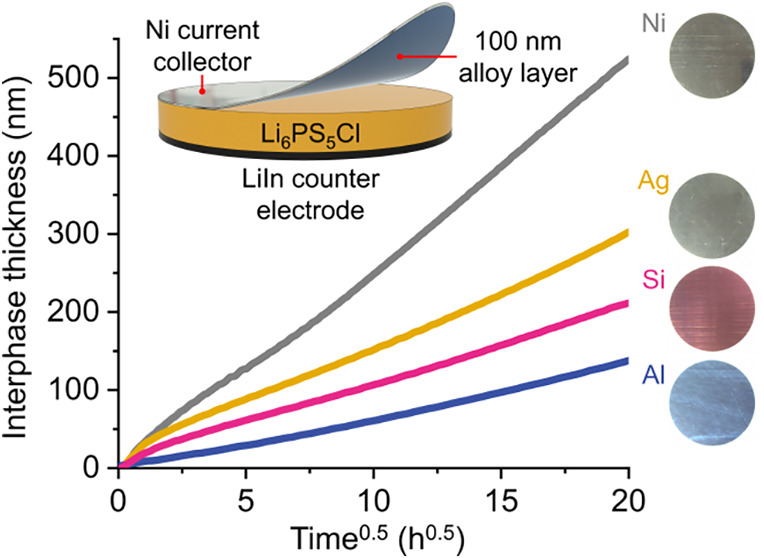

## Introduction

Solid-state electrolytes (SSEs) in solid-state batteries (SSBs) can provide various advantages over conventional liquid electrolytes in Li-ion batteries.^1,2^ High-capacity electrode materials such as Li metal and alloy anodes, which undergo large volume changes during Li insertion/extraction, are more compatible with SSEs than with flowable liquid electrolytes due to reduced electrode/electrolyte contact area and therefore diminished side reactions.^1,3,4^ However, contact loss caused by morphological changes of the electrode materials remains a challenge, requiring external stack pressures exceeding practically relevant values (typically 1–2 MPa for electric mobility applications).^4–9^ Moreover, although interphase growth is generally less severe than in liquid electrolytes since the SSE does not continually wet new electrode surface area exposed during volume changes, most SSEs are still thermodynamically unstable when in contact with negative electrode materials.^10–12^ This can result in a relatively thick (>300 nm) interphase layer at the SSE interface,^13–15^ and this interphase features different ionic conductivity and partial molar volume of Li compared to the parent SSE material. This can lead to increased cell resistance and nonuniform stress distribution across the SSE separator, which may contribute to SSB cell degradation.^14,16–18^

The mode of interphase growth at the interface between the SSE and the anode strongly depends on the composition and properties of the mixture of reduction products within the interphase layer.^1,19^ For instance, SSEs that contain certain cations can be reduced to form metallic phases within the interphase, which provide electronically conducting pathways that result in continuous reduction reactions at the interface.^17,20–22^ In contrast, the argyrodite Li_6_PS_5_Cl SSE, along with some oxide-type SSEs, typically form electronically insulating reduction products, thereby exhibiting relatively slow interphase growth kinetics in contact with Li metal.^13^ Li_6_PS_5_Cl is a particularly important SSE material due to its high ionic conductivity (>1 mS cm^–1^) and ease of processing into SSB cells.^23–25^ Although this material is kinetically stable in contact with Li metal, interphase growth still occurs and has implications for practical applications.^14^ Critically, while interphase growth has recently been investigated at the Li_6_PS_5_Cl|Li metal interface,^13–15,17,19,21,26–30^ there is limited understanding of interphase growth dynamics for other anode materials beyond Li.

Interphase growth kinetics and chemical composition have generally been investigated using thick, excess Li metal in direct contact with Li_6_PS_5_Cl in symmetric cell configurations.^17,30^ However, the inherent passivation layer present on thick Li metal can significantly influence interphase growth behavior in such cells.^30^ An anode-free SSB configuration offers a viable alternative for accurately observing and investigating interphase formation,^31^ as it enables the deposition of pure Li metal directly onto a current collector. Recently, a simple and precise electrochemical technique, coulometric titration time analysis (CTTA), was demonstrated to quantify the reduction reaction between Li_6_PS_5_Cl and Li metal.^13,14,26,28,32^ CTTA involves alternating titration steps, during which a small amount of Li is electrochemically deposited onto a current collector, and resting periods, during which chemical reduction reactions occur between the deposited Li and the Li_6_PS_5_Cl to form interphase and fully consume the Li. By monitoring the accumulated capacity titrated and then consumed over time, this method provides direct measurement of the amount of interphase that forms. This technique is widely applicable for understanding interface stability in a variety of systems.

Alloy anode materials comprise an alternative class of high-capacity anode materials that operate via the electrochemical formation of Li-rich alloys in host materials such as Si, Al, and In, and they hold great promise for SSBs.^33–35^ It is likely that alloy anodes exhibit different chemical stability at SSE interfaces compared to pure Li for a variety of reasons. The mismatch of Li chemical potential between Li metal and Li_6_PS_5_Cl, which drives interphase formation,^10^ is lowered when using alloy anodes because they typically exhibit positive electrode potentials vs. Li/Li^+^.^33,34^ Additionally, alloy anodes are host materials for Li, and the depletion of Li in the alloy host near the SSE interface during interphase growth may alter further Li transport or interphase reaction processes. Finally, Li filament growth, which can increase interfacial contact between Li metal and the SSE, can be mitigated by employing alloy anodes.^36,37^ These aspects suggest that interphase growth rates and interphase composition may be different when using alloy anodes compared to Li.

A few recent studies have focused on understanding the interphase between Si anodes and Li_6_PS_5_Cl.^38–40^ Using Si/Li_6_PS_5_Cl composite anodes, Huo et al. demonstrated that interphase growth plays a critical role in Si-based SSB performance.^39,40^ However, the interphases of other promising alloy anode materials for SSBs, such as Ag and Al,^4,6,36,37,41–44^ remain largely unexplored. Moreover, we have limited knowledge of the phenomena that influence and control interphase growth across different anode materials, including Si. Electrochemical impedance spectroscopy (EIS), commonly used for such analysis, provides incomplete information due to the presence of multiple contributors to interfacial impedance.^13,45^

Here, we present a systematic investigation of interphase growth when using various alloy anodes in cells with Li_6_PS_5_Cl SSE. A custom-fabricated, airtight SSB anvil cell was employed to perform CTTA on solid-state cells containing thin-film alloy layers (Ag, Al, Si, and Ge) supported on Ni current collectors, enabling the application of controlled stack pressures and temperatures. Different voltage responses were observed during titration and open-circuit rest steps for bare Ni and the alloy-coated Ni electrodes, indicating differences in interphase growth behavior. The corresponding accumulated capacities and extracted interphase thickness curves as a function of time revealed that the alloy-coated Ni electrodes exhibited less than half the amount of interphase growth compared to bare Ni electrodes at high stack pressures (8 and 50 MPa). In addition to the electrode potential (*i.e*., the thermodynamic driving force), we find that the interphase growth rates are also strongly affected by interfacial contact, which is governed by the mechanical properties of the anode material and the applied stack pressure. Due to differences in material mechanical properties and morphological evolution, alloy materials feature reduced interphase growth rates, whereas Li metal showed nonuniform 3D interphase growth under elevated stack pressures. This was further confirmed by time-of-flight secondary-ion mass spectrometry (ToF-SIMS) of interphase formed using bare Ni and Ag-coated Ni electrodes. This study quantifies interphase growth rates of technologically relevant alloy materials within SSBs and indicates that alloy anodes can enhance (electro)­chemical stability of the Li_6_PS_5_Cl interface compared to pure Li.

## Results and Discussion

CTTA measurements can exhibit strong dependence on the experimental conditions, including the titration current density, titration step capacity, temperature, temperature stability, applied stack pressure, and the choice of the current collector.^13,32^ Conducting such precise experiments using a standard SSB anvil cell inside a glovebox is not recommended, as slight temperature changes can lead to fluctuations in voltage curves and reaction rates.^46^ We thus designed and built airtight SSB anvil cells (Figure 1a) capable of preventing air ingress through a dual-locking mechanism utilizing O-rings and sealing caps (Figure 1b), while also allowing for the application of controlled stack pressures. These anvil cells were assembled and sealed inside an Ar-filled glovebox and subsequently transferred to a laboratory environmental chamber for CTTA testing at a constant temperature of 25 ± 0.5 °C, ensuring accurate and reproducible measurements. The chemical instability of typical Cu current collectors in contact with Li_6_PS_5_Cl can also influence CTTA results.^13^ Therefore, a Ni current collector was used as the working electrode to investigate interphase growth at the Li metal/Li_6_PS_5_Cl interface, as Ni is chemically intert toward both Li and Li_6_PS_5_Cl.^47,48^

**Figure 1:**
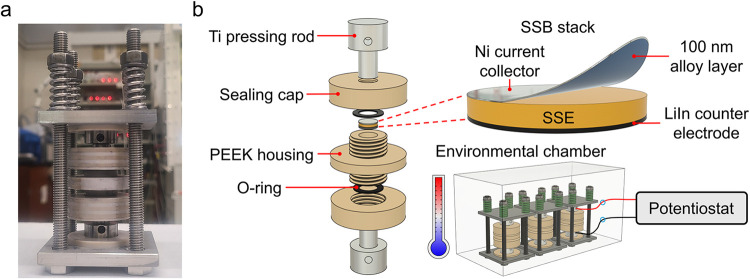
(a) Photograph of an airtight anvil SSB cell designed to prevent air ingress via a dual-locking mechanism while also enabling the application of controlled stack pressures. (b) Close-up, stacked view of an airtight anvil SSB cell, showing components including Ti pressing rods, sealing caps, the SSB stack, polyether ether ketone (PEEK) housing, and O-rings for airtight sealing. The SSB stack consists of a 100 nm alloy layer (Ag, Al, Si, or Ge) coated onto a Ni current collector, a Li_6_PS_5_Cl separator, and a LiIn counter electrode. The airtight anvil SSB cells were assembled inside an Ar-filled glovebox and subsequently transferred to an environmental chamber outside the glovebox for precise electrochemical measurements under constant temperature conditions.

Thin layers (100 nm) of selected Li-alloying elements (Ag, Al, Si, and Ge) were deposited onto Ni current collectors using magnetron sputtering for use as working electrodes to investigate interphase growth at alloy anode/Li_6_PS_5_Cl interfaces. These elements, commonly used as anode materials in SSBs, were selected based on their varying electrode potentials, with Ag having the lowest (closest to Li metal) and Ge the highest (Figure S1). Additionally, their distinct dealloying behaviors were considered; for instance, Al exhibits a two-phase reaction and Si features a single-phase reaction.^34^ Thin films were used instead of bulk foils or slurry-cast microparticle electrodes to minimize the diffusion of inserted Li into the remainder of the alloy material, which could occur with a typical thicker electrode. While sputtering metal layers directly onto the SSE surface is an alternative approach for studying interphase growth, we instead used coated Ni electrodes to better reflect the possible imperfect contact at the alloy anode/Li_6_PS_5_Cl interface and its effects on interphase growth. For the counter electrode, a LiIn alloy foil was used instead of the typical Li metal foil to mitigate the risk of Li penetration through the SSE separator during the long-term CTTA testing under high stack pressures.

The differences in interphase growth between Li metal and alloy anodes were first compared using bare Ni (on which Li deposits) and Ag-coated Ni (with which Li alloys) electrodes. To understand the nature of the CTTA experiment with the two electrodes, we paired CTTA with EIS. Impedance spectra were collected before the titration step and then periodically during the resting period after titrating both electrodes to 10 μAh cm^–2^ at a current density of 100 μA cm^–2^ (Figure 2). The subsequent rest period continued until the cell voltage returned to its initial value prior to titration. The time points at which impedance spectra were collected and plotted are indicated by circles on the potential plots (Figure 2a,c). Following titration onto the bare Ni electrode (Figure 2a), the potential during the resting period was constant at ∼0.005 V vs. Li/Li^+^ for 78 min, followed by a sharp increase in potential to ∼0.86 V vs. Li/Li^+^. As shown in the corresponding impedance spectra evolution (Figure 2b), the impedance spectra prior to titration displayed an extended tail at low frequencies, a characteristic feature typically associated with ion blocking electrodes.^49^ This behavior reflects the absence of Li transport across the Ni|Li_6_PS_5_Cl interface before titration. This tail disappeared after Li deposition, indicating a transition from an ion-blocking Ni|Li_6_PS_5_Cl interface to an ion-transferring Li|Li_6_PS_5_Cl interface. The spectrum remained similar during the period at which the open-circuit potential remained at ∼0.005 V vs. Li/Li^+^. As soon as the potential increased to ∼0.86 V vs. Li/Li^+^, however, the impedance spectra exhibited a sudden re-extension of the low-frequency tail, along with a gradual increase in both the angle and length of the tail with time. The re-extension indicates a transition back to an ion-blocking interface, signifying the complete consumption of the plated Li at the Ni|Li_6_PS_5_Cl interface during interphase formation. These results support the fundamental assumption of these CTTA experiments, which is that the electrochemically deposited Li is chemically consumed after deposition due to interphase formation during the time before the potential polarizes. We note that the sloping voltage curve beginning at ∼0.86 V vs. Li/Li^+^ is due to the gradual chemical delithiation of Li_3_P in the interphase to Li_*x*_P (*x* < 3), as identified and investigated in a recent study.^26^

**Figure 2:**
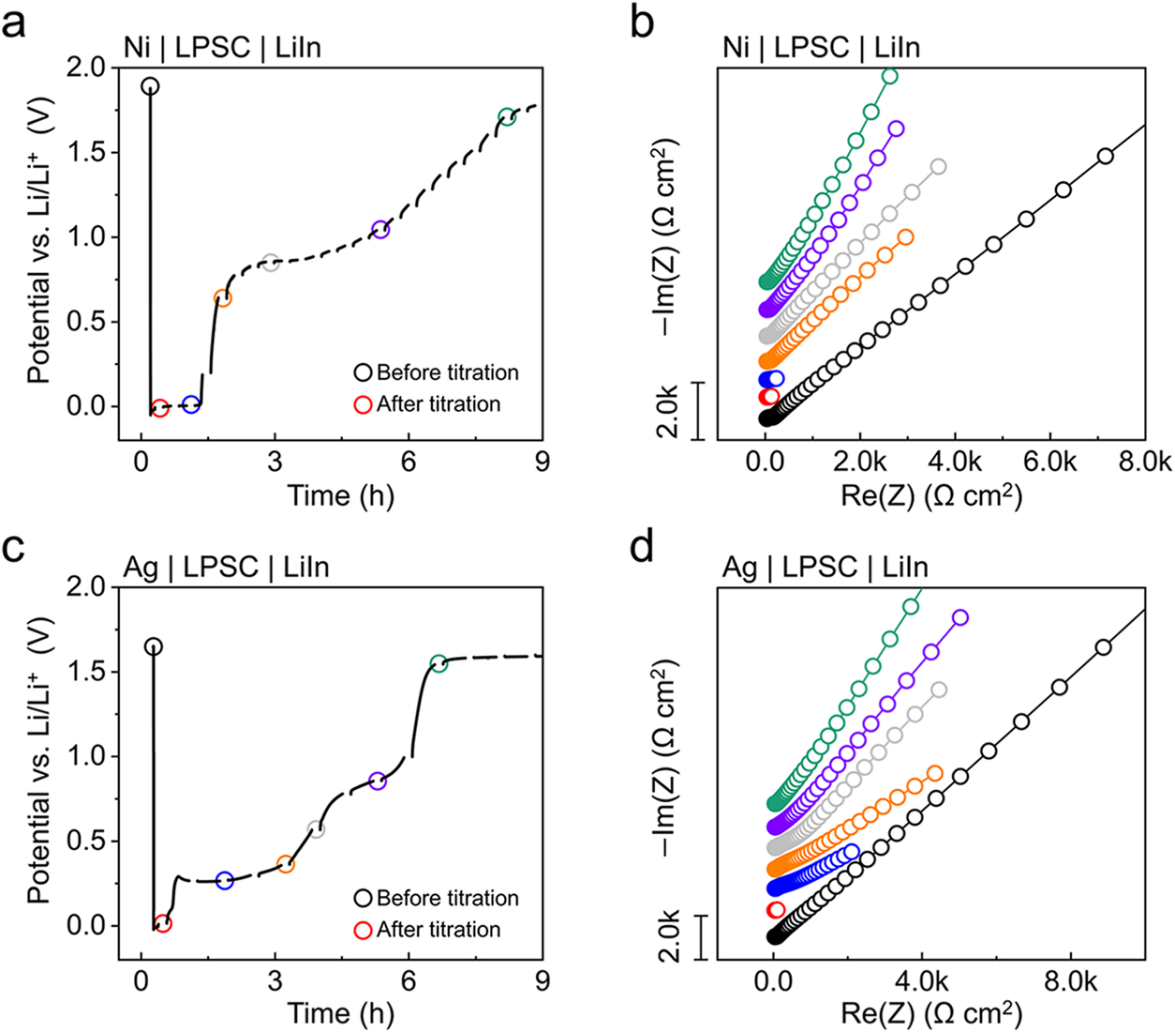
*In situ* EIS analysis during the first titration and open-circuit holding steps for cells with (a, b) bare Ni and (c, d) Ag-coated Ni electrodes. (a) Potential response and (b) corresponding impedance spectra evolution for a bare Ni electrode. (c) Potential response and (d) corresponding impedance spectra evolution for an Ag-coated Ni electrode. The time intervals during which the potential response was not recorded in (a) and (c) correspond to periods of EIS spectra measurements. The points at which the impedance spectra were collected and plotted in (b) and (d) are indicated by circles on the voltage curves. The titration step was performed at a current density of 100 μA cm^–2^ for 6 min, corresponding to a titration capacity of 10 μAh cm^–2^. The tests were conducted under a stack pressure of 8 MPa at 25 ± 0.5 °C in an environmental chamber.

The potential response of an Ag-coated Ni electrode (Figure 2c) showed a constant potential region for ∼ 3 h followed by a gradual potential increase to ∼0.86 V vs. Li/Li^+^ by ∼5 h. However, the potential of the initial constant region was ∼0.26 V vs. Li/Li^+^ instead of ∼0.005 V (as in the prior Li case). The potential of ∼ 0.26 V vs. Li/Li^+^ is attributed to the phase transformation of the Li–Ag alloy from the β phase to the α phase;^50^ this feature is also present in the first-cycle galvanostatic voltage curve of an Ag layer in a separate SSB half-cell experiment (Figure S1a). The impedance spectra of the Ag-coated Ni electrode (Figure 2d) showed a similar trend of sudden disappearance of the low-frequency tail after the titration. However, during the open-circuit hold portion of the experiment, the re-extension of the tail occurred progressively compared to the sharp re-extension observed when using the bare Ni electrode. This could be related to Li diffusion within the Li–Ag film, accompanied by a gradual change in the surface composition of the Li–Ag layer to become Li-deficient.

The duration of the constant-potential region during which the interphase was chemically formed was longer for the Ag-coated Ni electrode (at ∼0.26 V vs. Li/Li^+^) compared to the bare Ni electrode (at ∼0.005 V vs. Li/Li^+^), providing evidence of a slower interphase formation rate of the Li_6_PS_5_Cl in contact with the Li–Ag alloy compared to Li metal. Moreover, the sloping potential region beginning at ∼0.86 V vs. Li/Li^+^ (associated with Li_3_P delithiation) was shorter for the Ag-coated Ni electrode compared to the bare Ni electrode. Given the same titration capacity of 10 μAh cm^–2^, a comparable amount of interphase formation and, consequently, a similar duration of the sloping voltage region would be expected. This behavior suggests either compositional differences of the interphase layers formed in contact with the Li–Ag alloy vs. Li metal, or possible Li trapping within the Ag layer, resulting in a reduced quantity of formed interphase.

Figure 3 presents CTTA results for bare Ni and Ag-coated Ni electrodes tested under different stack pressures (1, 8, and 50 MPa). The CTTA was performed after an initial 5 min rest period, followed by repeated iterations of titration steps and resting periods. Titration was conducted for both electrodes with a capacity of 1.0 μAh cm^–2^ at a current density of 10 μA cm^–2^ in each step. The resting periods between titration steps were maintained until the open-circuit potential increased to 0.12 V vs. Li/Li^+^ (−0.50 V vs. LiIn/In) for bare Ni electrodes and 0.32 V vs. Li/Li^+^ (−0.30 V vs. LiIn/In) for Ag-coated Ni electrodes. These cutoff voltages were selected based on the *in situ* EIS results (Figure 2) to maintain the Li_3_P phase within the interphase layer and to avoid titration capacity loss due to cyclic lithiation of substoichiometric Li_*x*_P (*x* < 3).

**Figure 3:**
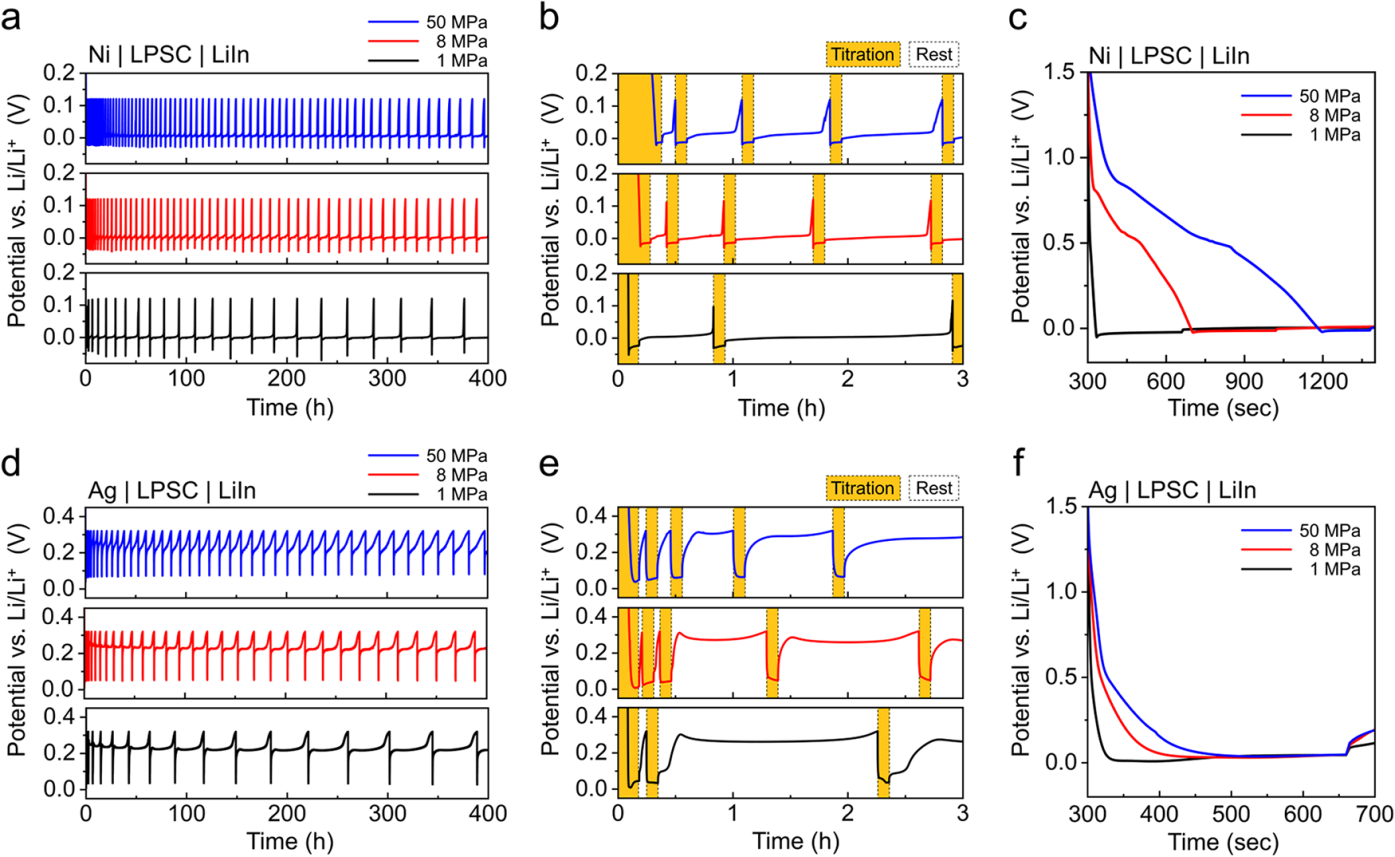
CTTA results for cells with (a–c) bare Ni and (d-f) Ag-coated Ni electrodes tested under different stack pressures of 1 MPa (black), 8 MPa (red), and 50 MPa (blue). (a) The potential during continuous titration and rest cycles over 400 h, (b) the potential over the initial 3 h, and (c) the first titration step for the bare Ni electrodes. (d) The potential during continuous titration and rest cycles over 400 h, (e) the potential over the initial 3 h, and (f) the first titration step for the Ag-coated Ni electrodes (100 nm thick). Titration steps were performed at a current density of 10 μA cm^–2^ for 6 min, corresponding to a titration capacity of 1.0 μAh cm^–2^. The cutoff voltages during resting steps were set to 0.12 V vs. Li/Li^+^ (−0.50 V vs. LiIn/In) for bare Ni and 0.32 V vs. Li/Li^+^ (−0.30 V vs. LiIn/In) for Ag-coated Ni electrodes. All tests were conducted at 25 ± 0.5 °C in an environmental chamber.

During 400 h of continuous titration/resting cycles, the bare Ni electrodes tested under different stack pressures showed noticeable differences (Figure 3a), displaying an increased number of titration steps and shorter resting periods with increasing stack pressure. This indicates that the interphase reaction was faster at higher stack pressures. As shown in the magnified potential curves during the initial 3 h of CTTA (Figure 3b), higher overpotentials were observed during titration (*i.e*., Li plating) at lower stack pressures, and this trend remained consistent throughout the 400 h of CTTA. For all stack pressures, the duration of the resting periods increased with the number of titration steps, indicating a slowing of the interphase formation rate as it grew thicker. This behavior is consistent with previous CTTA studies on anode-free SSB cells with stainless steel current collectors.^13,14,26,28,32^

Figure 3c shows the potential profiles during the first titration step for the bare Ni electrodes at three different stack pressures, where the Ni|Li_6_PS_5_Cl interface is free from any pre-existing interphase. These initial titration profiles exhibited strong dependence on stack pressure. At the lower stack pressure of 1 MPa, the increased overpotential resulted in a rapid transition to Li plating at an electrode potential below 0 V vs. Li/Li^+^. In contrast, at the higher stack pressures of 8 and 50 MPa, sloping profiles were observed, corresponding to the initial electrochemical decomposition of Li_6_PS_5_Cl to Li_*x*_P (*x* < 3), Li_2_S, and LiCl (at ∼0.85 V vs. Li/Li^+^), followed by full lithiation of Li_*x*_P to Li_3_P (at ∼0.55 V vs. Li/Li^+^).^26^ The electrochemical growth of the interphase during the initial titration step continued until a time of ∼1200 s at 50 MPa, at which point the electrode potential dropped below 0 V vs. Li/Li^+^ and Li plating began. Although this initial electrochemical formation of the interphase was observed at higher stack pressures, electrodes at these same stack pressures still exhibited more rapid chemical growth of the interphase during subsequent CTTA.

For the Ag-coated Ni electrodes, a noticeable increase of the intervals between titration steps was observed compared to bare Ni electrodes at all stack pressures, along with continuous dealloying of the Li–Ag alloy (Figure 3d). This suggests slower interphase formation on the Li–Ag electrodes. During the early stages of CTTA (Figure 3e), the potential curves showed a sharp increase to the cutoff voltage during the initial few resting periods. This behavior is attributed to insufficient lithiation of the Li–Ag alloy to the β phase to result in a constant region of ∼0.26 V vs. Li/Li^+^ during the resting periods. Interestingly, the Ag-coated Ni electrodes displayed different potential profiles during the initial titration step (Figure 3f) compared to the bare Ni electrodes (Figure 3c). The duration of electrochemical Li_6_PS_5_Cl decomposition was substantially suppressed at stack pressures of 8 and 50 MPa, indicating reduced electrochemical interphase formation on the Li–Ag alloy during the initial titration step. In addition, the initial titration potential profiles observed for the Ag-coated Ni electrodes exhibited less dependence on stack pressure and lacked sharp nucleation overpotentials for Li deposition characteristic of the bare Ni electrodes.^36^ The reduced nucleation overpotential for the Ag layer suggests a lower energy barrier for Li nucleation and dissolution within the Ag host structure, thereby reducing the likelihood of Li_6_PS_5_Cl decomposition at the SSE interface.

CTTA was also conducted for other thin-film alloy anode materials, including Al-, Si-, and Ge-coated Ni electrodes (Figure 4). The cutoff potential during the resting periods was set to 0.62 V vs. Li/Li^+^ (0.0 V vs. LiIn/In) for all three electrodes, corresponding to a potential near the onset of the sloping region associated with Li_3_P delithiation, as observed from *in situ* EIS experiments (Figure 2a,c). A clear dip was observed in the potential curves of Al-coated Ni electrodes during each titration step (Figure S2a,b), which arises from the nucleation of the LiAl phase on the Al surface.^51^ The repeated formation of a flat-potential region at ∼0.42 V vs. Li/Li^+^ during each resting period over 400 h of CTTA (Figure 4a), followed by a rapid potential increase at the end of this region, is associated with the chemical dealloying of LiAl to form the pure Al phase due to reaction with Li_6_PS_5_Cl to form interphase (Figure S1b).

**Figure 4:**
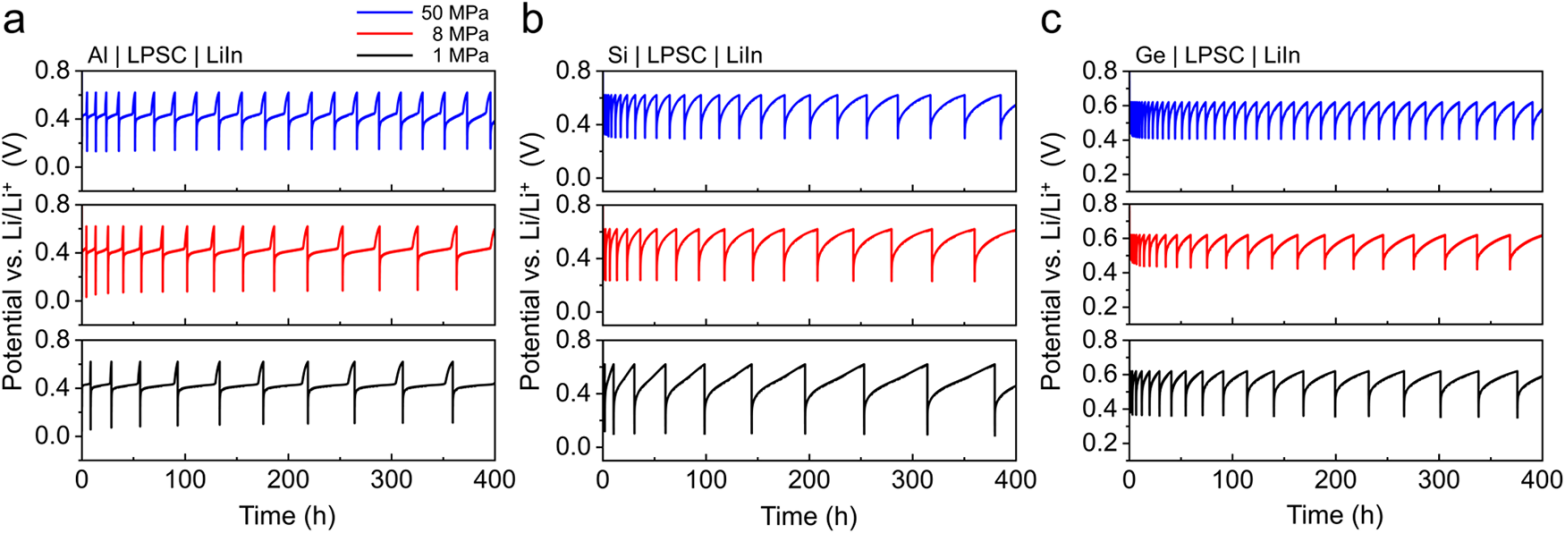
CTTA results for cells with various electrodes tested under different stack pressures of 1 MPa (black), 8 MPa (red), and 50 MPa (blue). Potential profiles during continuous titration and rest cycles over 400 h for the (a) Al-, (b) Si-, and (c) Ge-coated Ni electrodes (all 100 nm thick). Titration steps were performed at a current density of 10 μA cm^–2^ for 6 min (corresponding to a titration capacity of 1.0 μAh cm^–2^). The cutoff voltage during resting steps were set to 0.62 V vs. Li/Li^+^ (0 V vs. LiIn/In). All tests were conducted at 25 ± 0.5 °C in an environmental chamber.

The shapes of the CTTA curves were different for the Si- and Ge-coated Ni electrodes (Figure 4b,c). Although the curves showed repeated increases to the cutoff potential during the resting periods, the sharp voltage polarization observed in the Al-coated Ni electrodes was absent. This is likely due to the different dealloying behavior of amorphous Li_*x*_Si and Li_*x*_Ge, which undergo a single-phase delithiation mechanism characterized by increasing electrode potential,^52^ which is different from the constant electrode potential associated with a two-phase reaction with a sharp reaction front in the case of LiAl (Figure S1b–d).^51^ As a result, it is difficult to deconvolute the potential signals during the resting periods that are associated with the dealloying of Li_*x*_Si or Li_*x*_Ge from the onset of Li_3_P delithiation. Although direct evidence of dealloying features associated with Li_6_PS_5_Cl reduction reactions is lacking for the Si and Ge cases, the substantial voltage rise from the equilibrium potential and the increase in the duration of resting periods (a typical characteristic of Li_6_PS_5_Cl interphase growth) clearly indicate interphase formation in contact with Li_*x*_Si and Li_*x*_Ge layers.

Overall, the Al-, Si-, and Ge-coated Ni electrodes exhibited a clear trend of higher rates of interphase growth with higher stack pressures, as well as noticeably longer resting periods for each stack pressure compared to those of bare Ni electrodes. In other words, these results indicate slower interphase formation on all of these alloys than for pure Li electrodes. As shown in Figure S2, the alloy layers also exhibited only minor extents of electrochemical interphase formation during the initial lithiation step compared to pure Li, even at the high stack pressure of 50 MPa.

A direct comparison of the interphase growth rates between bare Ni and alloy-coated Ni electrodes was conducted by plotting the accumulated consumed capacity over 400 h of CTTA for different stack pressures (Figure 5a). Averaged data from three different experiments were used for each curve, for a total of 45 experiments of 400 h each (see Supporting Information, Figures S3–12 for associated data sets). For Li and the alloy materials, the accumulated capacity curves are approximately dependent on the square-root of time, indicative of diffusion-controlled interphase growth. As expected from the CTTA voltage curves in Figures 3 and 4, the accumulated capacity curves for the bare Ni electrodes displayed the greatest sensitivity to stack pressure, with significantly accelerated interphase growth observed at 50 MPa. In contrast, alloy layers (Ag, Al, Si, and Ge) exhibited less dependency on stack pressure, as well as consistently lower accumulated capacity values across all stack pressure levels compared to bare Ni electrodes. The real impedance in the high frequency region was greater for the Ni electrode compared to the Ag-coated electrode after 400 h of CTTA at 50 MPa (Figure S13), suggesting degradation of Li transport due to more substantial interphase growth at the Li_6_PS_5_Cl/Ni electrode interface.^8,36^

**Figure 5:**
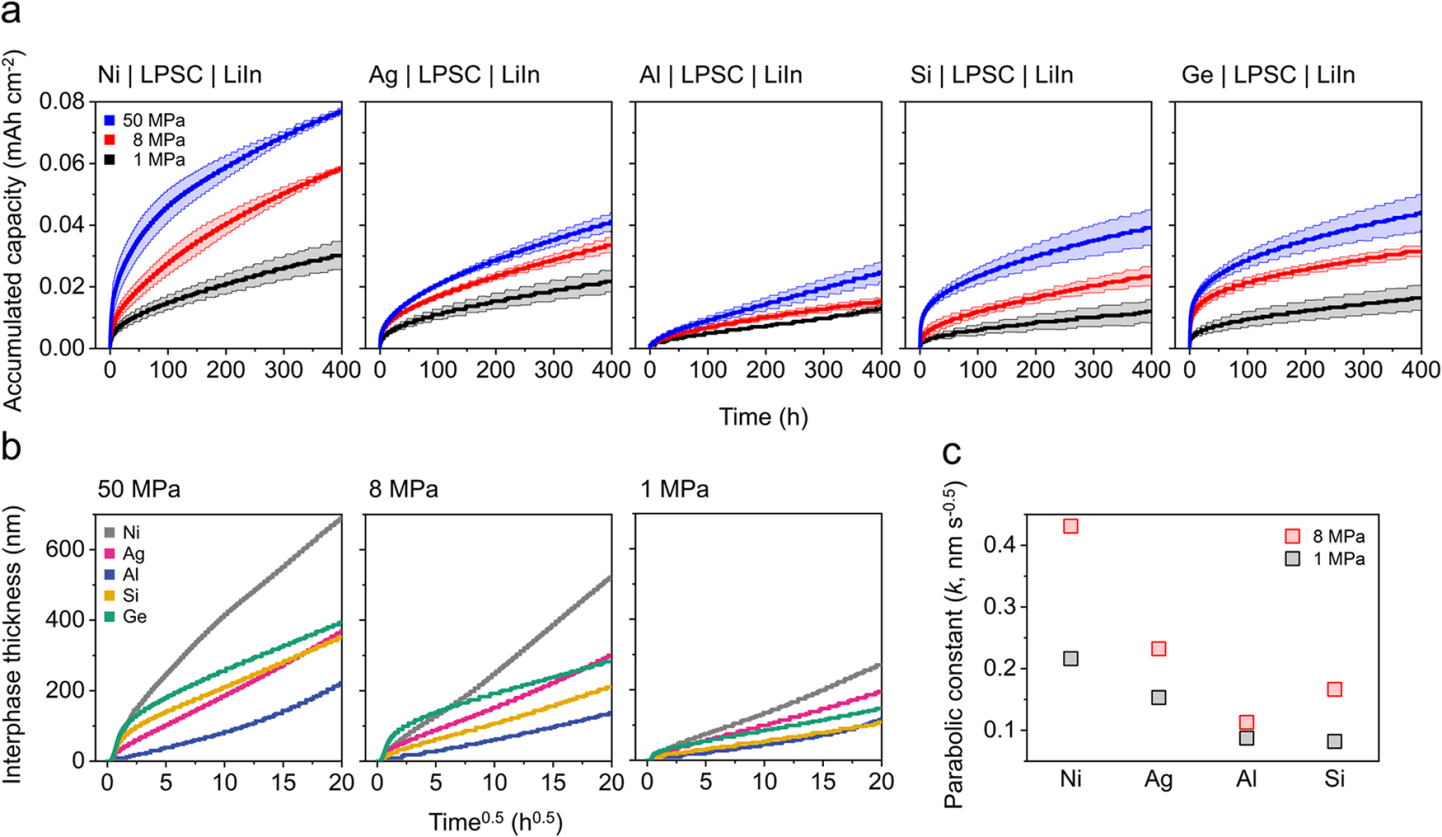
(a) Accumulated capacity curves over 400 h of CTTA measurements for cells with bare Ni electrodes (*i.e*., Li deposition), and Ag-, Al-, Si-, and Ge-coated Ni electrodes, tested under stack pressures of 1, 8, and 50 MPa. Each curve represents the average of three replicate cells, with shaded regions indicating the standard deviation. (b) Calculated interphase thickness as a function of the square root of time (h^0.5^) for the different electrode materials plotted at different stack pressures. (c) Comparison of the parabolic rate constants extracted from the slopes of the plots shown in panel (b). The parabolic rate constants were calculated for electrode materials (Ni, Ag, Al, Si) and applied stack pressures (1 and 8 MPa) that exhibited linear behavior in panel (b).

To better understand and compare the interphase growth rate on different anode materials, the interphase thickness was calculated from the average accumulated capacity in Figure 5a. The accumulated capacity was assumed to be entirely consumed by the reduction reaction with Li_6_PS_5_Cl. The interphase was also assumed to be a dense, homogeneous, two-dimensional layer. The associated parameters, including the molar volumes of interphase species, were adopted from a previous study.^13,14^ Figure 5b presents the resulting interphase thickness curves for bare Ni and alloy layer-coated electrodes at different stack pressures. For bare Ni electrodes under 1 and 8 MPa, the calculated interphase thickness displays a linear dependence on the square root of time (h^0.5^). In contrast, the curve for 50 MPa is sublinear, suggesting three-dimensional interphase growth.^53^ The differences in interphase growth thickness on Ni electrodes during CTTA as a function of stack pressure clearly indicate that morphology differences during Li plating influence the interphase growth at the Li metal/Li_6_PS_5_Cl interface. A higher interfacial contact area between Li metal and Li_6_PS_5_Cl, likely resulting from dendritic Li plating under elevated stack pressures, may have facilitated increased interfacial surface area formation. Conversely, reduced contact area between Li metal and Li_6_PS_5_Cl due to irregular and spatially distributed Li plating at 1 MPa stack pressure may have contributed to limited interfacial growth.^31,54^

As expected from the accumulated capacity curves in Figure 5a, the interphase thickness was calculated to be thinner on the electrodes with alloy layers, with greater thickness differences between Li and the alloys at higher stack pressures. Most of the alloys exhibit close to linear dependence of thickness on the square root of time. However, deviations from the linear dependence on the square root of time are also observed for some alloy layer-coated electrodes, which are particularly evident during the early stages of CTTA for the Ge-coated Ni electrodes at all stack pressures and for Si-coated Ni electrodes at 50 MPa. This deviation arises from the continuous titration behavior observed during the initial stage (<1 h) of CTTA, where a portion of the titration capacity is consumed by alloying reactions to form additional Li_*x*_Si or Li_*x*_Ge alloy phases instead of reacting to form interphase (Figure S2c,e). As the electrode potentials decrease to levels sufficiently below the cutoff, the Si- and Ge-coated Ni electrodes can effectively initiate interphase formation reactions with Li_6_PS_5_Cl within the potential range, which then continue throughout the entire 400 h of CTTA. A different deviation behavior was observed for the Al-coated Ni electrodes at 50 MPa, characterized by a slight increase in slope during the later stage of CTTA. This is likely attributed to diffusional Li trapping within the electrode, caused by low Li diffusivity of the pure Al phase, which promotes voltage polarization to the cutoff potential.^34^ Additionally, morphological changes of Al toward the later part of CTTA may have exacerbated this effect.^4,42^

A Wagner-type diffusion model was applied to the interphase growth curves in Figure 5b that closely follow a parabolic trend (i.e., those that show a linear dependence on the square root of time). The curves for bare Ni, as well as Ag-, Al-, and Si-coated Ni electrodes, at 1 and 8 MPa stack pressures (a total of eight curves) were linearly fitted to extract parabolic rate constant (*k*) values (see Figure S14 for fitted plots). These values are compared across different anode materials and stack pressures in Figure 5c. The *k* value of 0.431 nm s^–0.5^ for Li grown on the bare Ni electrode at 8 MPa closely matches the previously reported value of 0.46 nm s^–0.5^ for Li metal.^14^ The *k* values at 8 MPa of Ag-coated Ni (*k* = 0.232 nm s^–0.5^), and Al-coated Ni electrodes (*k* = 0.113 nm s^–0.5^) are lower than that for pure Li. Given that the parabolic rate constant is dependent on the thermodynamic driving force (*i.e*., the chemical potential difference of Li between the anode and the SSE), the increasing electrode potentials from bare Ni to Ag to Al observed during the titration steps (Figure S15) likely contribute to the decreasing trend in parabolic rate constant.

While this result provides evidence that alloy anodes can exhibit reduced interphase growth rates due to their higher electrode potentials relative to Li, further analysis indicates that the interphase growth rate is complicated by other factors. As an example, the Si-coated Ni electrode shows a parabolic rate constant of 0.166 nm s^–0.5^ at 8 MPa, which is higher than the Al-coated Ni electrode despite the similar electrode potential (Figure S15). Additionally, the parabolic rate constant was reduced for all electrodes as the stack pressure decreased from 8 to 1 MPa, which suggests that interfacial contact may play a role.

The diffusion-controlled solid-state reaction model is governed by Fick’s law, in which the flux of Li through the interphase layer is determined by the chemical potential gradient of Li between two boundaries (*i.e*., the electrode/interphase and interphase/SSE interfaces). However, in SSB systems, the extent of interfacial contact at the electrode/interphase interface is another critical factor that can influence the Li flux and, consequently, alter the resulting parabolic rate constant values. The interfacial contact is strongly influenced by the mechanical properties and morphological evolution of alloy anodes during lithiation. The ductile-to-brittle transition of Al and the associated crack formation can lead to loss of interfacial contact.^4,34,42^ In contrast, Si typically exhibits the opposite behavior,^55^ forming a dense Li_*x*_Si phase with reduced crack volume upon lithiation.^4,7^ Ag is known for its good adhesion to the SSE, suggesting the formation of a conformal and stable contact at the interface during lithiation.^56^ These differences in mechanical behavior are a potential reason that Al exhibits the slowest interphase growth, as well as the minimal change in its parabolic rate constant upon reducing the stack pressure.

The interphase growth behavior during CTTA measurements, where the interphase evolution is monitored during open-circuit holding steps, is somewhat different from that occurring during continuous cell cycling. To examine this aspect, SSB full cells with Li metal or alloy anodes were cycled under a stack pressure of 5 MPa for comparison. LiNb_0.5_Ta_0.5_O_3_ (LNTO)-coated LiNi_0.6_Mn_0.2_Co_0.2_O_2_ (NMC622) cathodes were used for all experiments. Figure S16 shows the first cycle charge curves of SSB full cells employing a Ni current collector, Li metal, and different alloy anodes. The alloy anodes include a Si microparticle electrode, a 1 μm-thick sputtered Si electrode, and a 1 μm-thick Ag film electrode. The Li foil cell shows a sloping plateau beginning at ∼ 1.8 V, which was absent in data from the other cells. This plateau indicates electrochemical decomposition of Li_6_PS_5_Cl at the Li metal/SSE interface; the extended decomposition in the Li foil cell is likely due to the greater extent of interfacial contact of soft Li metal under this stack pressure (5 MPa). The increase in the bulk resistance even after 50 cycles in the Li metal full cell indicates that the SSE decomposition occurs continuously during cycling (Figure S17c). These results suggest that SSE degradation proceeds not only through self-discharge or calendar aging but also during SSB operation.

The differences in interphase growth of Li_6_PS_5_Cl in contact with Li metal and the Ag alloy were further characterized using ToF-SIMS chemical analysis. Bare Ni and Ag-coated Ni electrodes were examined from cells after 400 h of CTTA at 50 MPa stack pressure (Figure 6). This highest stack pressure was selected for investigation since it is associated with the largest interphase thickness differences (Figure 5b). Upon completion of the CTTA measurements, the airtight SSB anvil cells were returned to the glovebox, and the SSB stacks were removed for *ex situ* characterization. During the extraction of the stack from the PEEK housing, the Ni current collector was completely detached from SSE pellet, while the Ag layer remained adhered to the SSE surface for the Ag-coated sample. ToF-SIMS was subsequently conducted on the Ag-coated SSE surface and on the SSE surface that was originally in contact with the bare Ni current collector (Figure 6a).

**Figure 6:**
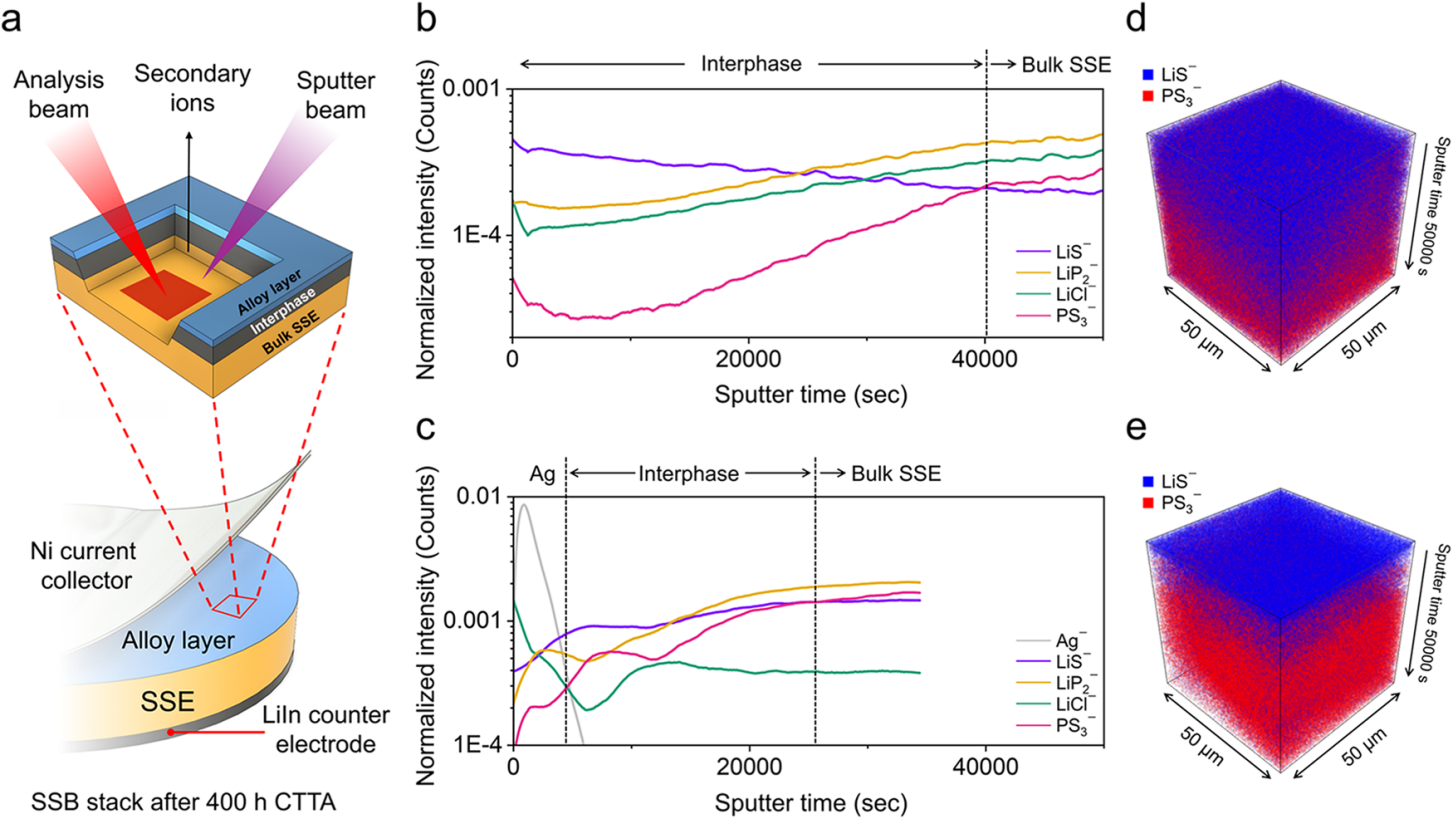
Interface characterization using ToF-SIMS for cells with bare Ni and Ag-coated Ni electrodes. (a) ToF-SIMS measurements were conducted on samples from SSB stacks subjected to 400 h of CTTA under 50 MPa stack pressure. During the SSB stack removal process, the Ag layer detached from the Ni current collector and remained in contact with the SSE. (b, c) ToF-SIMS depth profiles of the electrode/SSE interface from SSB stacks with (b) bare Ni and (c) Ag-coated Ni electrodes. (d, e) 3D reconstructed ToF-SIMS depth profile volume images of the electrode/SSE interface from SSB stacks with (d) bare Ni and (e) Ag-coated Ni electrodes.

Figure 6b shows the ToF-SIMS depth profile of the material stack from the bare Ni electrode upon which Li was deposited. The LiS^–^, LiP_2_^–^, and LiCl^–^ signals correspond to molecular fragments from the interphase layer, while the PS_3_^–^ signal originates from the bulk SSE. The PS_3_^–^ signal showed a gradual increase and stabilization at sputter time of ∼ 40,000 s, indicating the boundary between the interphase layer and the bulk SSE. The region before the PS_3_^–^ signal stabilized corresponds to the interphase region; in this region, the LiS^–^ signal steadily decreased, while the LiP_2_^–^ and LiCl^–^ signals increased and then stabilized. This indicates a heterogeneous interphase microstructure for Li metal consisting of Li_2_S-rich and Li_3_P/LiCl-rich regions within the interphase layer, consistent with observations from previous studies.^13,15,28^ The ToF-SIMS depth profile of the material stack from the Ag-coated Ni electrode cell revealed a similar heterogeneous interphase microstructure, but with significantly reduced interphase thickness (Figure 6c). The boundary between the Ag layer and the interphase was identified at a sputter time of ∼6000 s, and stabilization of the PS_3_^–^ signal (indicative of the bulk SSE) occurred at a sputter time of ∼26,000 s. Thus, the Ag-coated Ni electrode featured an interphase with approximately half the thickness of the bare Ni electrode, which is consistent with the trends in accumulated capacity and calculated interphase thickness shown in Figure 5a,b. The obtained ToF-SIMS depth profiles were compared in three dimensions, with the LiS^–^ signal representing the interphase region and the PS_3_^–^ signal representing the bulk SSE. The 3D mapping of the depth profile at the interface of the bare Ni electrode cell (Figure 6d) revealed a much thicker and nonuniform distribution of the LiS^–^ signal compared to the uniformly distributed LiS^–^ signal observed at the interface of the Ag-coated Ni electrode cell (Figure 6e). This further indicates the beneficial effect of the alloy anode in suppressing filamentary Li growth, which can lead to three-dimensional interphase growth at high stack pressures.

For additional understanding of the interphase, scanning electron microscopy (SEM) and X-ray photoelectron spectroscopy (XPS) were carried out to further elucidate the surface morphology and chemical environment at the alloy anode/SSE interface. In contrast to the ToF-SIMS analysis, these measurements were conducted on both the electrode surface (the alloy layer delaminated from the SSE during SSB stack removal) as well as the corresponding SSE surface. Figure S18 shows SEM images of the electrode surfaces before and after 400 h of CTTA under 50 MPa stack pressure. The uneven spatial distribution in the energy dispersive spectroscopy (EDS) map of the S signal (Figure S18b) for the bare Ni electrode after CTTA indicates significant morphological changes in the SSE at the interface, as confirmed by SEM images of Li_6_PS_5_Cl (Figure S19a,b). These changes are attributed to nonuniform interphase growth, which led to localized adhesion of the interphase to the Ni current collector. The alloy anodes, on the other hand, displayed uniform EDS maps of the S signal (Figure S18d,f,h), and the corresponding SSE surface exhibited minimal morphological changes (Figure S19a,c).

Figure S20 shows XPS S 2p spectra from both sides of the extracted interfaces (the SSE side and the electrode side) before and after 400 h of CTTA under 50 MPa. The increase in peak intensity at ∼160 eV in the S 2p spectra after CTTA compared to the Li_6_PS_5_Cl reference, observed on both the electrode and SSE surfaces, indicates the formation of Li_2_S.^57^ This is further supported by the peak shift toward lower binding energy observed in the Li 1s spectra (Figure S21) after CTTA,^13,14^ consistent with the results obtained from ToF-SIMS analysis. A surface oxide layer was detected from Al and Si 2p spectra (Figure S22c,d) from the Al- and Si-coated Ni electrodes. In particular, the Al 2p spectra after CTTA showed a peak shift of the Al metal peak toward higher binding energy. The intermediate peak located between Al oxide and Al metal peaks corresponds to Al–S species.^58^ These observations suggest that the inherent surface oxide layer on alloy anodes, or newly formed interphase species distinct from conventional interphase components (Li_2_S, Li_3_P, or LiCl), may contribute to the suppressed interphase growth of alloy anodes compared to Li metal.

## Conclusions

This study demonstrates that various alloy anode materials (Ag, Al, Si, and Ge) exhibit lower rates of interphase growth and therefore enhanced interfacial stability when in contact with the widely used Li_6_PS_5_Cl electrolyte in SSBs. After 400 h of CTTA, the alloy-coated Ni electrodes exhibited less than half of the accumulated capacities associated with interphase growth compared to those of bare Ni electrodes under high stack pressures. Aluminum showed the lowest interphase growth rates of all the alloy materials. From direct comparison of the calculated interphase thickness as a function of the square root of time, we find that interphase growth rate is influenced not only by the electrochemical behavior of the electrode materials but also by their mechanical properties and the applied stack pressures. In general, lower stack pressures lead to reduced interfacial contact, which slows interphase growth; these effects likely depend on the mechanical properties of the lithiated alloys. In contrast to the extensive increase in interfacial contact area for Li metal at high stack pressures leading to 3D interphase growth, the 2D interface of alloy anodes exhibited suppressed and more stable interphase growth. The reduced and uniform interphase growth rates of alloy anodes were further supported by ToF-SIMS depth profiling and corresponding 3D maps of the SSE interface. Furthermore, the measured growth rates can be used to predict interphase growth in other electrode geometries, such as composite electrodes.

From these precise measurements, we estimate a 10% loss of SSE material from a 20 μm-thick pure Li_6_PS_5_Cl separator (in other words, 2-μm thick interphase formation) within eight months in Li metal-based SSBs under 8 MPa stack pressure. In contrast, the same thickness of interphase is projected to grow over ∼10 years when using an Al electrode under the same stack pressure. This order-of-magnitude improvement highlights the potential for alloy anode materials for achieving longer-term interfacial stability in SSBs. Although these alloy anodes suppress interphase growth compared to Li metal in contact with Li_6_PS_5_Cl, the extent of interphase growth is still too high to maintain an internal resistance below 40 Ω cm^2^, a reported threshold resistance for practical SSB operation.^59^ This emphasizes the need for continued R&D efforts to further improve SSE stability through the integration of interlayers or novel electrode architectures.

## Methods

### Electrode Preparation

For the fabrication of alloy-coated Ni electrodes, 1 μm or 100 nm-thick layers of Ag, Al, Si, and Ge were deposited onto 10 μm-thick Ni current collectors (MSE Supplies) using an Amod 060 Physical Vapor Deposition (PVD) system (Angstrom Engineering). The deposition chamber was evacuated to a base pressure of <1 × 10^–6^ mTorr, after which Ar was introduced and maintained at ∼1 mTorr. During deposition, the Ag target was used in pulsed DC mode at a power density of 22 W in^–2^ (22% of the maximum DC power), with the output increased at a ramp rate of 20% min^–1^. The Al target was used in pulsed DC mode at 75 W in^–2^ (50% of maximum DC power), also with 20% min^–1^ ramp rate. The Si target was used in pulsed DC mode at 2 W in^–2^ (10% of maximum DC power), with a slower ramp rate of 5% min^–1^. The Ge target was used in RF mode at 1 W in^–2^ (5% of maximum RF max power), with the output increased at a ramp rate of 0.5% min^–1^. LiIn counter electrodes were fabricated by preparing Li (MSE Supplies) and In foils in a 1:1 atomic ratio (Li:In). The In foils were prepared by cold-rolling In pellets (Kurt J. Lesker) using an electric Durston rolling mill. The Li and In foils were then stacked and mixed via an accumulative roll bonding process inside an Ar-filled glovebox. The resulting LiIn counter electrodes can prevent Li penetration-induced cell short circuiting at high stack pressures while providing a stable electrode potential of 0.62 V vs. Li/Li^+^.^4,34^ The cathode composite consisted of LiNi_0.6_Mn_0.2_Co_0.2_O_2_ (NMC622), Li_6_PS_5_Cl, and vapor grown carbon fiber (VGCF). To mitigate interfacial degradation with the SSE, NMC622 was surface-modified with a LiNb_0.5_Ta_0.5_O_3_ (LNTO) coating. The coating precursor was prepared by dissolving stoichiometric amounts of niobium ethoxide, tantalum butoxide, and lithium acetate in anhydrous ethanol, into which NMC622 powder was dispersed via sonication. After solvent removal under vacuum, the resulting powder was annealed at 450 °C to form the LNTO layer. The final cathode composition was 70 wt % coated NMC622, 27.5 wt % Li_6_PS_5_Cl, and 2.5 wt % VGCF, homogenized by dry ball milling in a ZrO_2_ jar. Si particulate electrodes were prepared using microscale silicon particles (Alfa Aesar), *N*-methyl-2-pyrrolidone (Sigma-Aldrich) solvent, and 0.1 wt % polyvinylidene difluoride binder. The slurry was cast onto a Ni current collector (identical to that used for alloy layer preparation) using a doctor blade. The cast electrodes were dried under vacuum at 100 °C overnight to remove the solvent.

### Cell Assembly

SSB cells were assembled inside an Ar-filled glovebox using bare Ni and alloy-coated Ni electrodes as working electrodes, Li_6_PS_5_Cl as the SSE separator, and LiIn foil as the counter electrode. The 10 mm-diameter working electrodes were prepared using a disk cutter (MTI). 90 mg of ultrafine Li_6_PS_5_Cl powder (∼1 μm particle size, MSE Supplies) was poured into a polyether ether ketone (PEEK) die (inner diameter: 10 mm) and uniaxially pressed to a pressure of ∼250 MPa for 5 min using Ti plungers. Subsequently, the bare Ni or alloy-coated Ni electrode and LiIn counter electrode were added and further pressed to ∼375 MPa. The sealing caps were then tightened inside the Ar-filled glovebox to prevent air ingress prior to transferring the cells to the laboratory environmental chamber for CTTA testing. For SSB full cell assembly, the SSE separator was prepared by uniaxially pressing the Li_6_PS_5_Cl powder at 250 MPa. The composite cathode layer was then applied and further densified at 375 MPa. A 50 μm Li foil (MSE Supplies) was placed on the counter electrode side and pressed at 50 MPa to ensure conformal interfacial contact. For cells with alloy anodes, the cathode and alloy anode, separated by the SSE layer, were copressed at 375 MPa.

### Electrochemical Testing

The assembled cells were sandwiched between steel stack plates, and stack pressures of 1, 8, and 50 MPa were applied by tightening nuts at each corner using a digital torque wrench. The applied stack pressures were calibrated using a pressure sensor prior to testing. All electrochemical tests were conducted in an environmental chamber (Espec, BTU-133) maintained at 25 ± 0.5 °C. Galvanostatic discharge/charge tests for 1 μm alloy-coated Ni electrodes were performed using an Arbin Instruments LBT21084uC-5 V1A battery cycler. The half cell tests were conducted at a current density of 10 μA cm^–2^ within a voltage range of 0 to 1.0 V vs. Li/Li^+^ (−0.62 to 0.38 V vs. Li/Li^+^), under a stack pressure of 50 MPa. EIS measurements were carried out using a BioLogic SP-200 potentiostat for cells with bare Ni and 100 nm Ag-coated Ni electrodes under a stack pressure of 8 MPa. Impedance spectra were collected before and after the titration step, as well as periodically during the open-circuit hold step. The titration step was performed at a current density of 100 μA cm^–2^ for 6 min (titration capacity of 10 μA cm^–2^). EIS measurements were recorded over a frequency range of 2 MHz to 2 Hz. CTTA measurements were conducted using a Squidstat potentiostat (Admiral Instruments). The titration steps were carried out at a current density of 10 μA cm^–2^ for 6 min (titration capacity of 1.0 μA cm^–2^). The cutoff voltages during open-circuit holding steps were set to 0.12 V vs. Li/Li^+^ (−0.50 V vs. LiIn/In) for bare Ni electrodes, 0.32 V vs. Li/Li^+^ (−0.30 V vs. LiIn/In) for 100 nm Ag-coated Ni electrodes, and 0.62 V vs. Li/Li^+^ (0 V vs. LiIn/In) for 100 nm Al-, Si-, and Ge-coated Ni electrodes. Note that the electrode potential (vs. Li/Li^+^) was approximated by adding 0.62 V to the measured cell voltage during both open-circuit hold and titration steps. It should also be noted that the accumulated capacity comparisons shown in Figure 5 are based on the CTTA results using different cutoff potentials for each electrode material. The SSB full cells were cycled using a Neware battery testing system at a current density of 0.25 mA cm^–2^ under a stack pressure of 5 MPa, within a voltage range of 2.0 to 4.1 V.

### CTTA Measurement and Data Analysis

CTTA is an electrochemical technique primarily used to analyze electrolyte stability. After titration, the cell reaches equilibrium, and the time required for this equilibrium to be disrupted by side reactions during open-circuit holding steps serves as the key parameter. The resulting trend of accumulated capacity over time provides valuable insight into the interphase growth mode and kinetics, as well as overall electrolyte stability, enabling direct comparison among different electrolyte-electrode combinations.

The accumulated amount of titrated Li (*n*_Li_; mol cm^–2^) was calculated from the accumulated capacity (mAh cm^–2^) using Faraday’s constant (*F*). A complete chemical reaction between Li (either deposited Li or alloyed Li-M alloy) and SSE was considered, as shown below:

$${\mathrm{Li}}_{6}{\mathrm{PS}}_{5}\mathrm{Cl}+8\mathrm{Li}\to 5{\mathrm{Li}}_{2}S+{\mathrm{Li}}_{3}P+\mathrm{LiCl}$$

Assuming a dense, homogeneous, two-dimensional interphase layer, the interphase thickness (*d*_interphase_) was calculated using the following equation:

$$d_{\mathrm{interphase}}=\frac{n_{\mathrm{Li}}}{8}\cdot \left(5V_{{\mathrm{Li}}_{2}S}+V_{{\mathrm{Li}}_{3}P}+V_{\mathrm{LiCl}}\right)$$

The molar volumes of interphase species used in the calculation were 27.5 cm^3^ mol^–1^ for Li_2_S, 19.8 cm^3^ mol^–1^ for Li_3_P, and 35.0 cm^3^ mol^–1^ for LiCl.^14^ The averaged interphase thickness values from three different experiments were linearly fitted with a relative standard error of slope less than 0.1%.

### Materials Characterization

After 400 h of CTTA measurements under 50 MPa, the SSB stacks with bare Ni and Ag-coated Ni electrodes were removed from the PEEK housing inside an Ar-filled glovebox and transferred to an IONTOF 5–300 system for ToF-SIMS analysis. For depth profiling, a Cs^+^ beam (150 × 150 μm^2^, 2 kV, 10 nA) was used for sputtering, and a Bi cluster primary ion (50 × 50 μm^2^, 25 kV) was used to scan the central area of the sputtered crater for secondary ion detection. Measurements were conducted in negative polarity mode, and a flood gun was used for charge neutralization. Data acquisition was performed with 3 shots per pixel over a 128 × 128 pixel raster. The depth profiles and 3D maps were reconstructed with SurfaceLab 6.2 software. The resulting depth profile data were smoothed using a Savitzky-Golay filter. The surface SEM images and EDS maps of electrode and SSE surfaces were obtained using a Thermo Fisher Helios 5CX SEM. Both the alloy layer that was delaminated from the SSE surface and the corresponding SSE were transferred to the SEM chamber, with air exposure of a few seconds. XPS characterization was performed using a Thermo K-Alpha XPS system equipped with an Al Kα source. Samples were prepared in an Ar-filled glovebox, following the same procedure as for SEM sample preparation, and they were transferred to the XPS instrument using a vacuum transfer holder to avoid exposure to air. The XPS spectra were collected using a 400 μm spot size with a base pressure less than 2.5 × 10^–7^ mbar. The sample surface was gently etched prior to spectrum acquisition, and surface charging was compensated with a flood gun.

## Supplementary Material



## Data Availability

The source data for this paper can be found at 10.5281/zenodo.17886673

## Notes

First-cycle galvanostatic voltage curves of SSB half cells; Voltage curves during the early stages of CTTA; Voltage curves during continuous titration and rest cycles over 400 h; Accumulated capacity curves over 400 h of CTTA; EIS spectra collected before and after 400 h of CTTA; Calculated interphase thickness plotted as a function of square root of time (h^0.5^); Electrode potentials plotted as a function of accumulated capacity (mAh cm^–2^) during continuous titration steps; First charge curves of SSB full cells employing different negative electrodes; Galvanostatic cycling of an SSB full cell using 50 μm Li foil as the negative electrode; SEM images and corresponding EDS maps of electrodes before and after 400 h of CTTA; SEM images of Li_6_PS_5_Cl pellet before and after 400 h of CTTA; XPS S 2p and Li 1s spectra of Li_6_PS_5_Cl reference material, as well as spectra after 400 h of CTTA from both the electrode side and the SSE side of different electrodes; XPS spectra of pristine electrodes, as well as the electrode materials after 400 h of CTTA, collected from both the SSE side and the electrode side (PDF)
