# Supplementary material for: Tracking Interphase Growth at Alloy Anode Interfaces in Sulfide Solid-State Batteries
Source: J Am Chem Soc. 2025 Dec 31;148(1):581–92. doi: 10.1021/jacs.5c15251 (PMC12814352; doi:10.1021/jacs.5c15251)
Supplement: Supplementary file 1 [file ja5c15251_si_001.pdf]

Supporting Information

Tracking interphase growth at alloy anode interfaces in sulfide solid-state batteries

Won Joon Jeong[1], Douglas Lars Nelson[1], Congcheng Wang[2], Sun Geun Yoon[2], Donghyeok Roh[2], Elif Pınar Alsaç[2], Kelsey Anne Cavallaro[2], Lincoln Crowe[3], Matthew T. McDowell[1,2]*

[1]School of Materials Science and Engineering, Georgia Institute of Technology, Atlanta, GA, 30332, USA

[2]George W. Woodruff School of Mechanical Engineering, Georgia Institute of Technology, Atlanta, GA, 30332, USA

[3]School of Chemical and Biomolecular Engineering, Georgia Institute of Technology, Atlanta, GA, 30332, USA

*Corresponding Author: mattmcdowell@gatech.edu

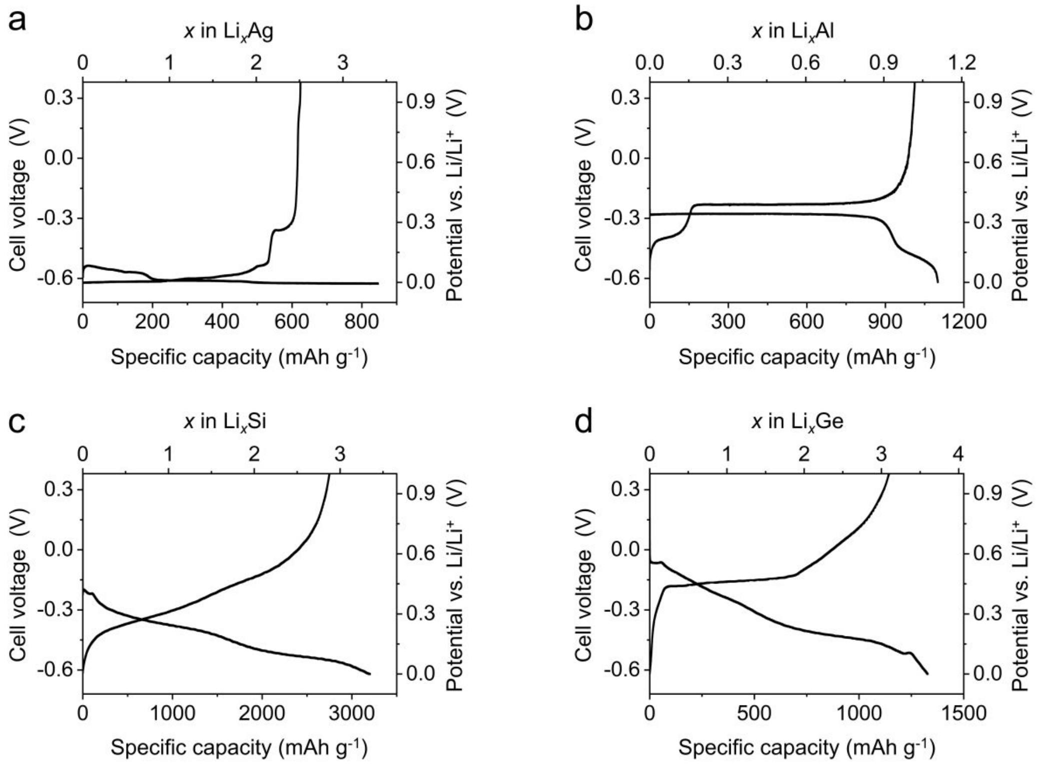

Figure S1. First-cycle galvanostatic voltage curves for 1 µm-thick alloy anodes in SSB half cells using Li_6PS_5Cl SSE and LiIn counter electrodes. (a) Ag-, (b) Al-, (c) Si-, and (d) Ge-coated Ni electrodes. All cells were tested under a stack pressure of 50 MPa and a current density of 10 µA cm^{-2} using airtight anvil cells at 25 °C inside an environmental chamber.

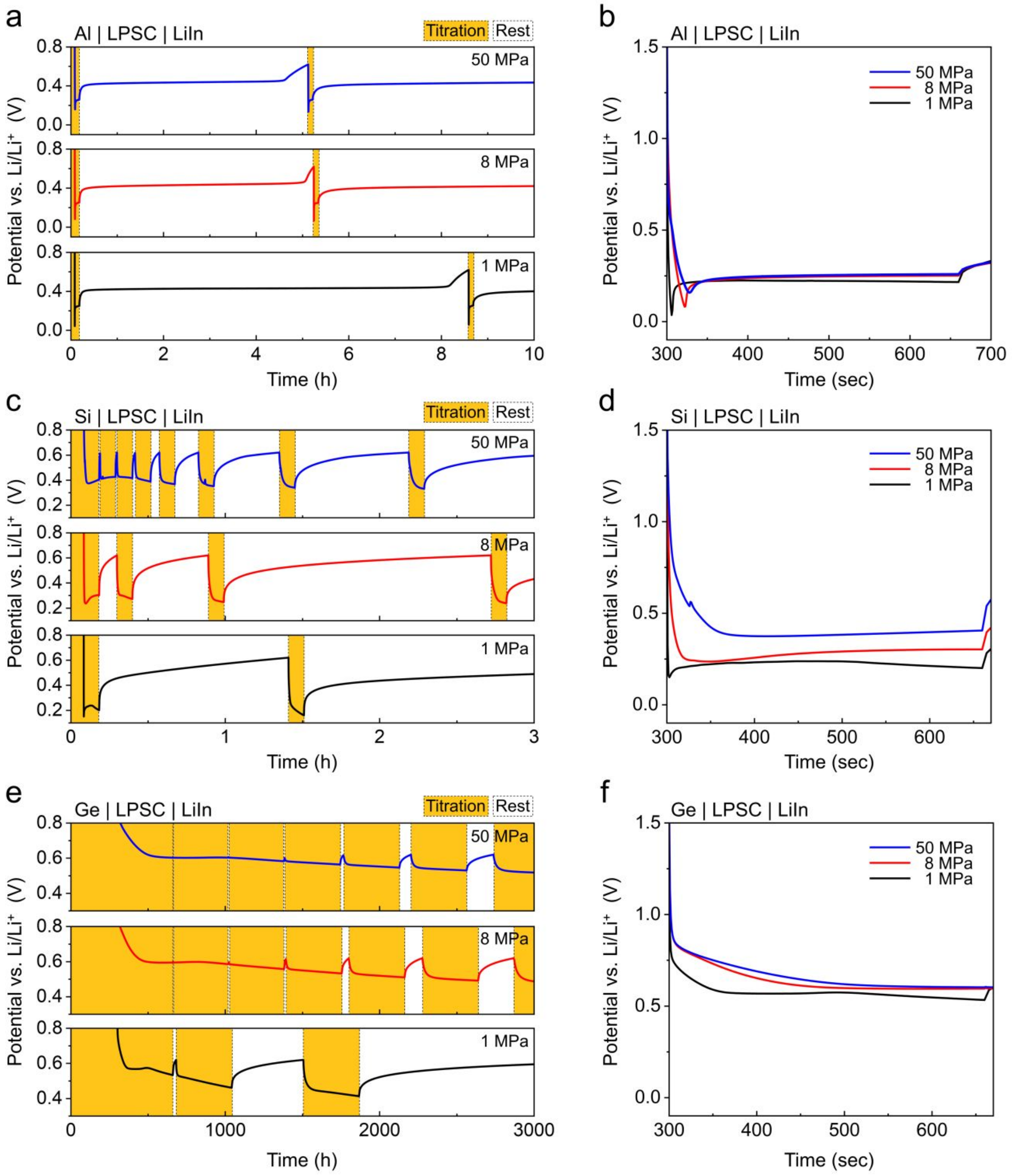

Figure S2. Voltage curves during the early stages of CTTA and the first titration step following a 300 s rest period prior to the start of CTTA for alloy layer-coated Ni electrodes tested under different stack pressures. (a, b) 100 nm Al-, (c, d) Si-, and (e, f) Ge-coated Ni electrodes.

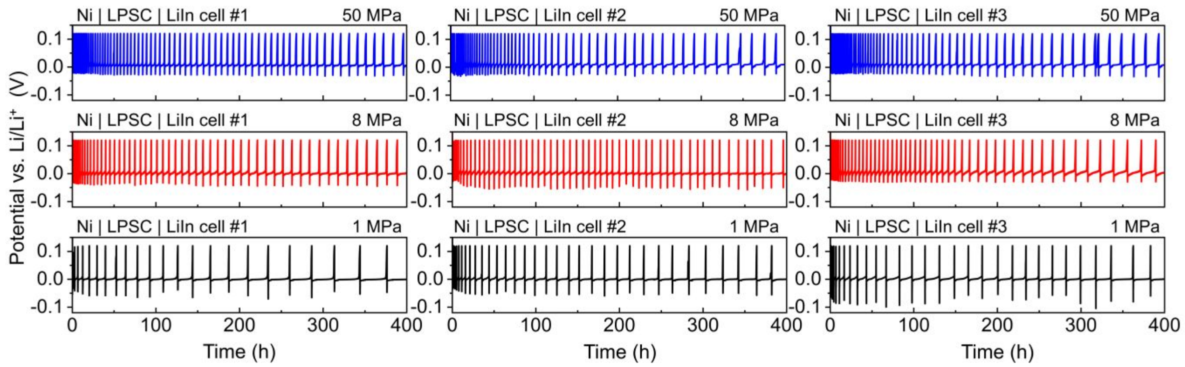

Figure S3. Voltage curves during continuous titration and rest cycles over 400 h for bare Ni electrodes tested under different stack pressures. Tests were performed three times using airtight SSB cells for each stack pressure.

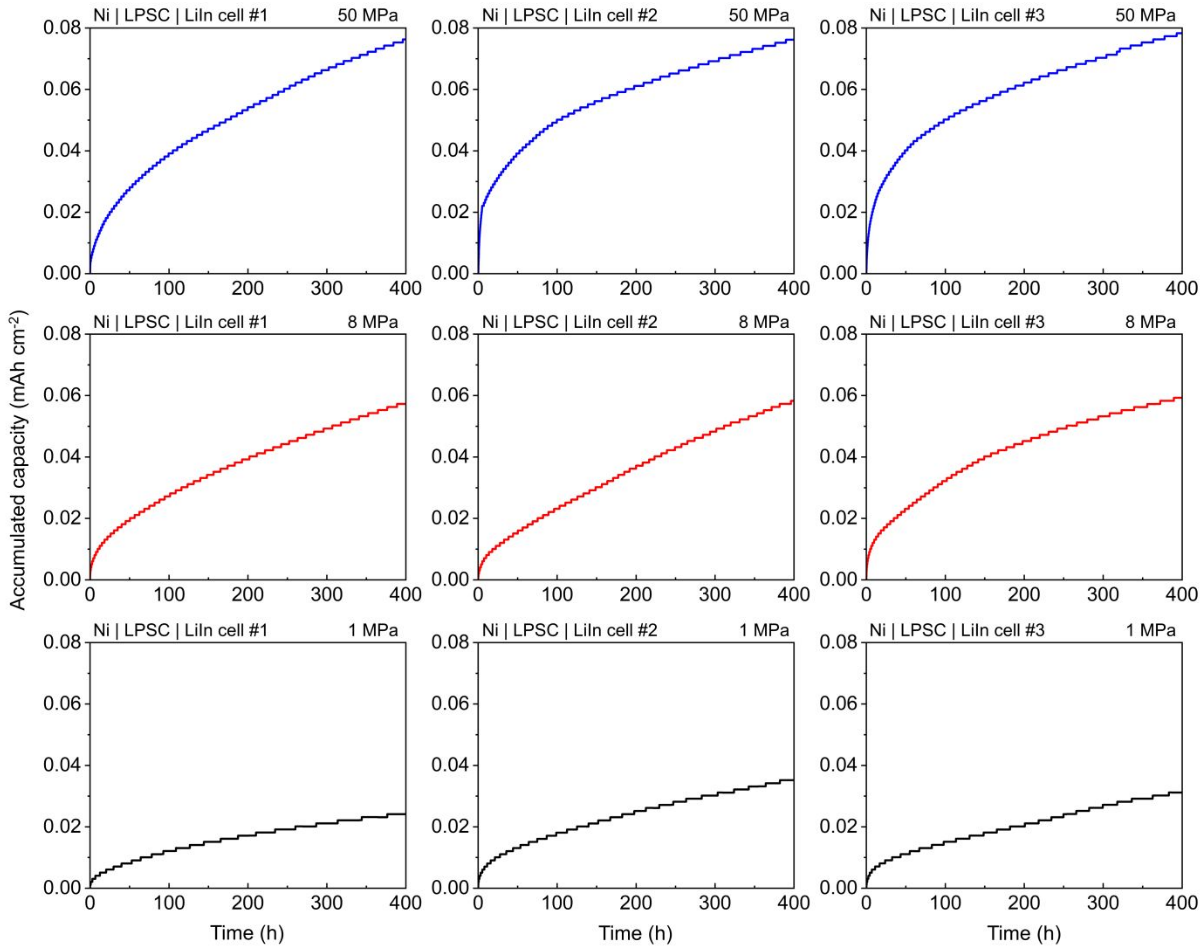

Figure S4. Accumulated capacity curves over 400 h of CTTA for the bare Ni electrodes at different stack pressures.

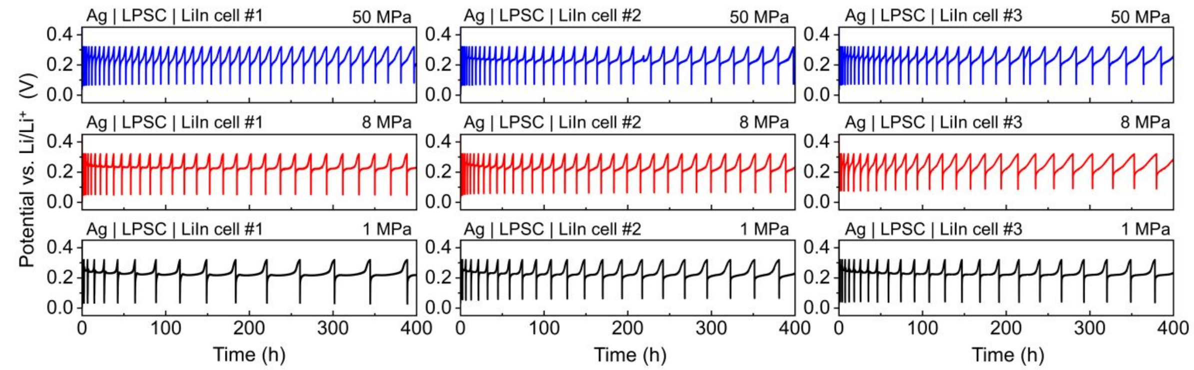

Figure S5. Voltage curves during continuous titration and rest cycles over 400 h for 100 nm Ag-coated Ni electrodes tested under different stack pressures. Tests were performed three times using airtight SSB cells for each stack pressure.

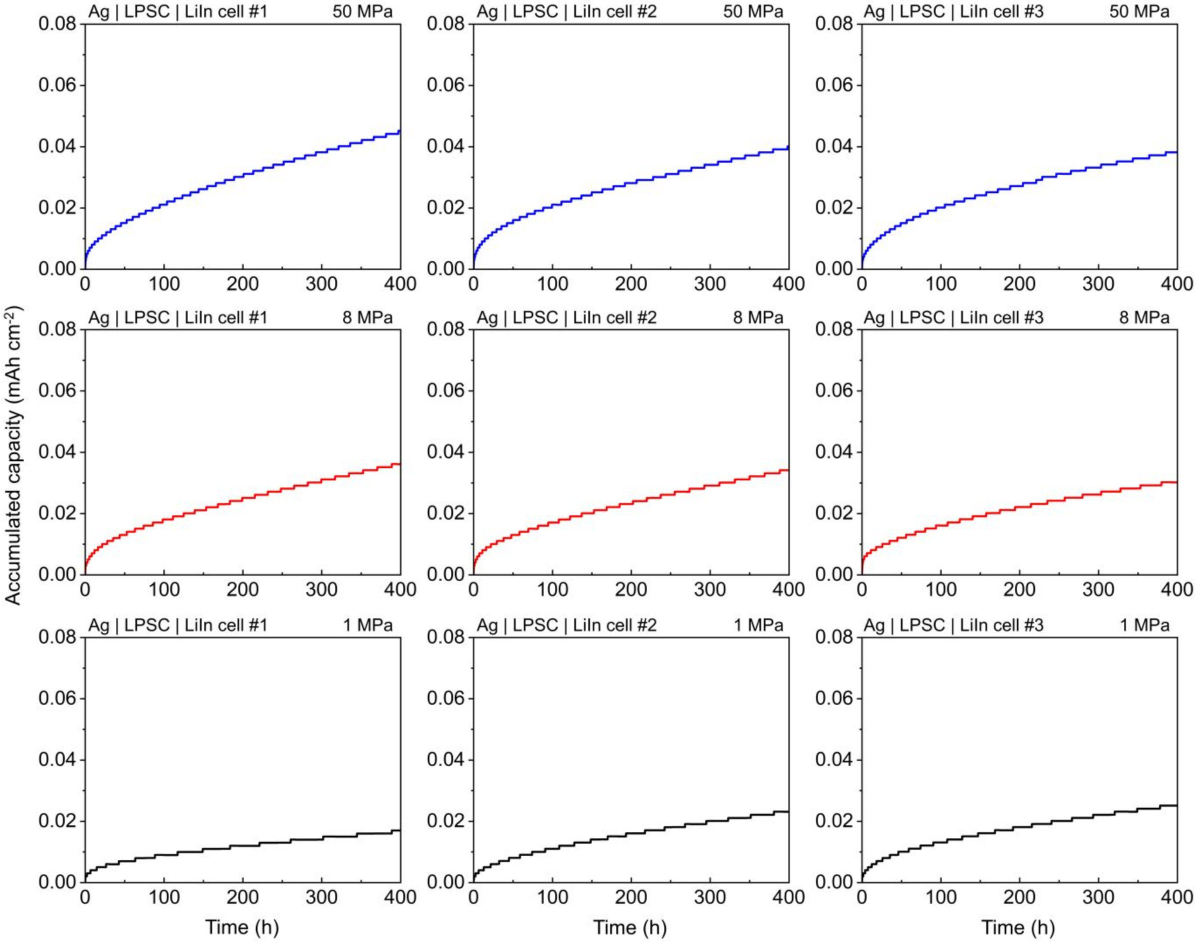

Figure S6. Accumulated capacity curves over 400 h of CTTA for 100 nm Ag-coated Ni electrodes tested under different stack pressures.

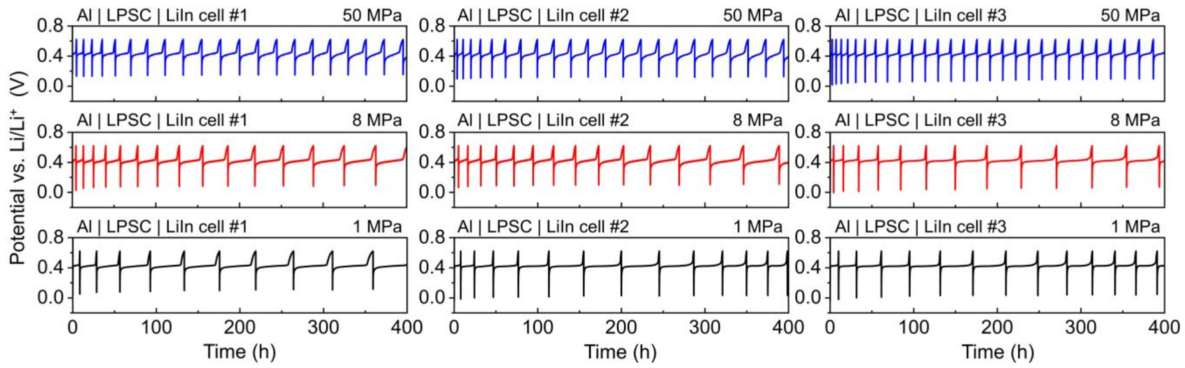

Figure S7. Voltage curves during continuous titration and rest cycles over 400 h for 100 nm Al-coated Ni electrodes tested under different stack pressures. Tests were performed three times using airtight SSB cells for each stack pressure.

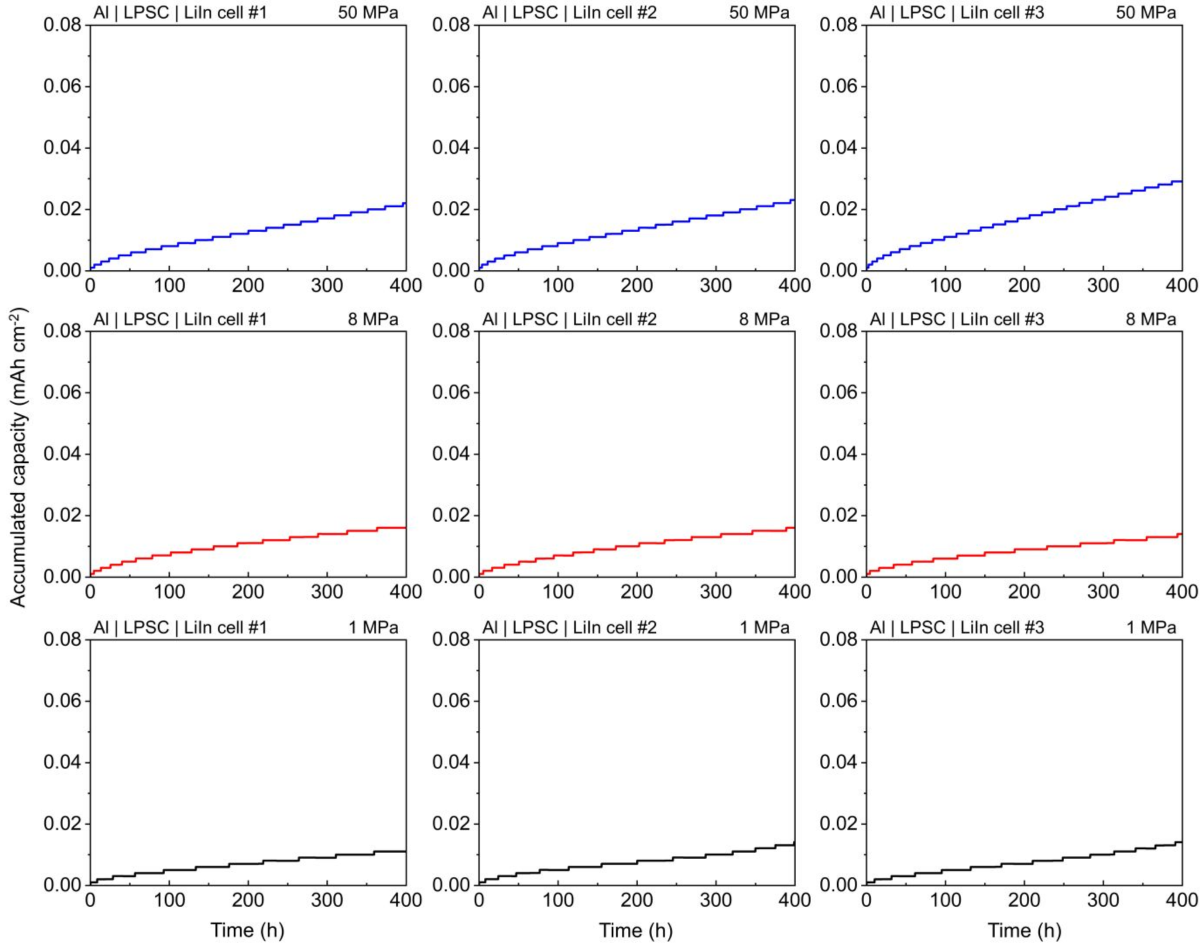

Figure S8. Accumulated capacity curves over 400 h of CTTA for 100 nm Al-coated Ni electrodes tested under different stack pressures.

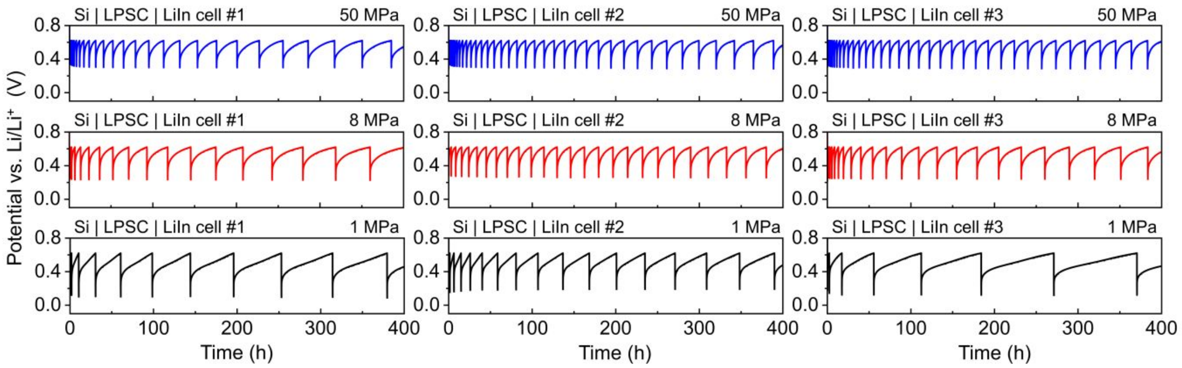

Figure S9. Voltage curves during continuous titration and rest cycles over 400 h for 100 nm Si-coated Ni electrodes tested under different stack pressures. Tests were performed three times using airtight SSB cells for each stack pressure.

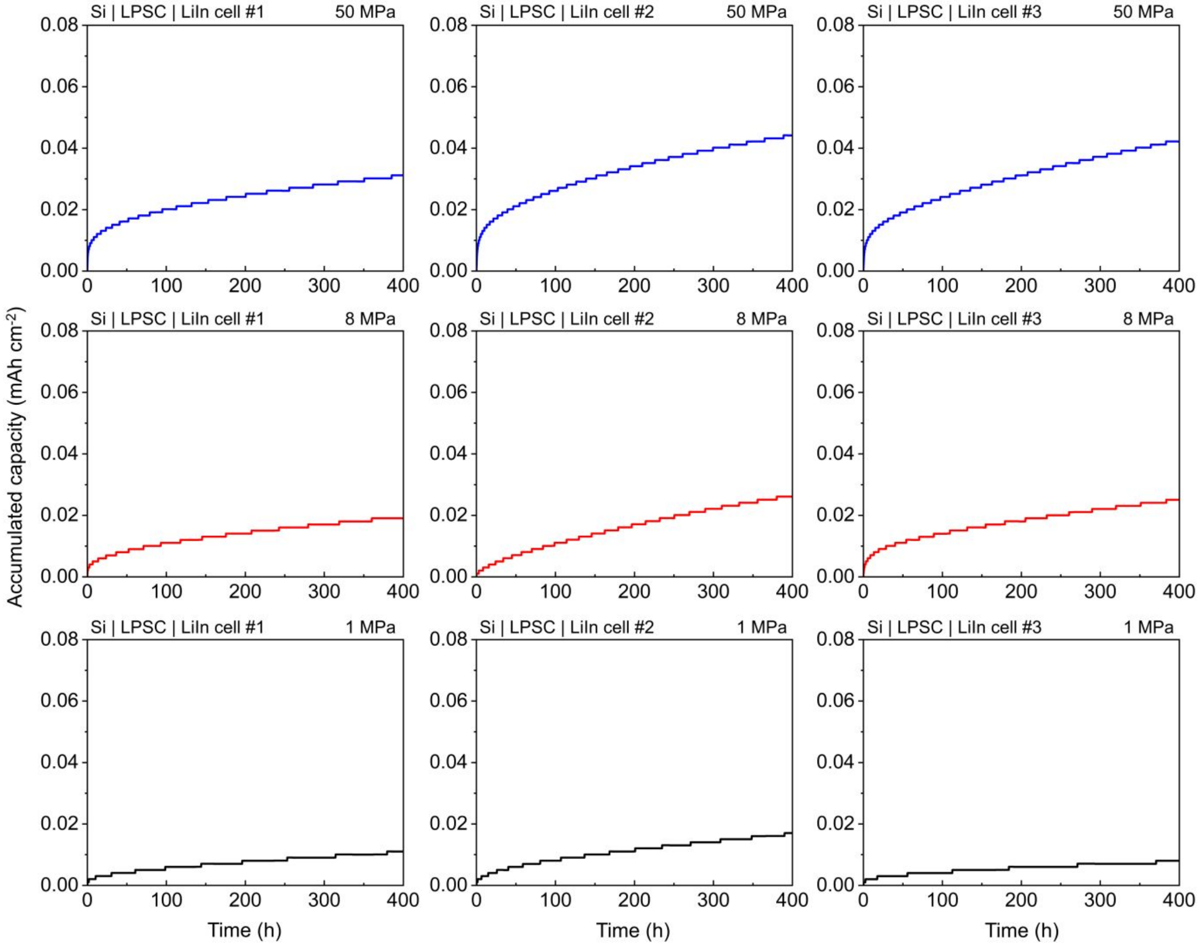

Figure S10. Accumulated capacity curves over 400 h of CTTA measurements for 100 nm Si-coated Ni electrodes tested under different stack pressures.

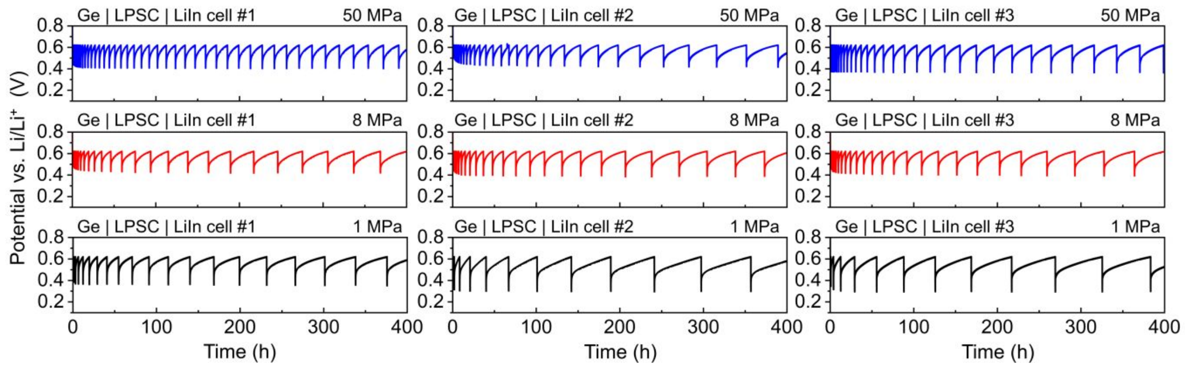

Figure S11. Voltage curves during continuous titration and rest cycles over 400 h for 100 nm Ge-coated Ni electrodes tested under different stack pressures. Tests were performed three times using airtight SSB cells for each stack pressure.

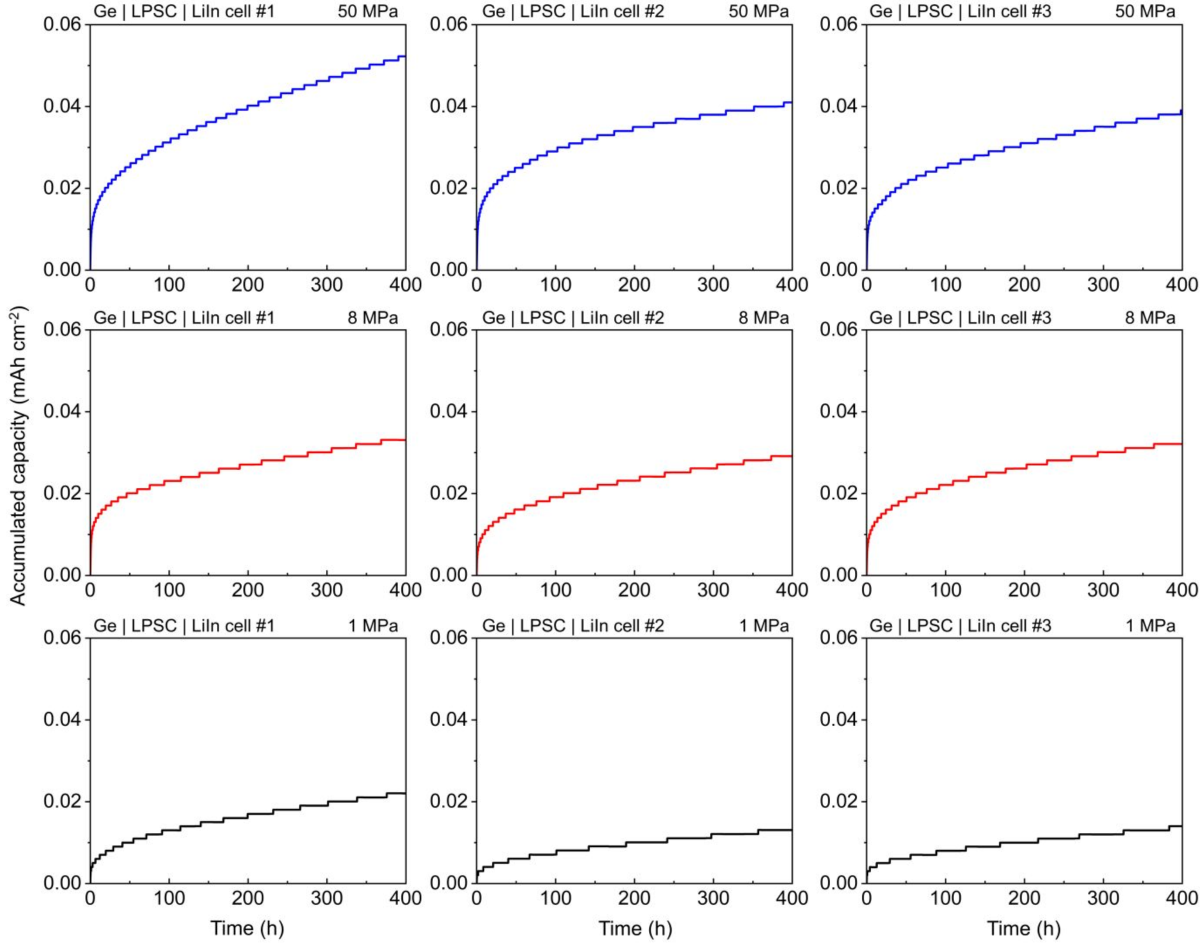

Figure S12. Accumulated capacity curves over 400 h of CTTA measurements for 100 nm Ge-coated Ni electrodes tested under different stack pressures.

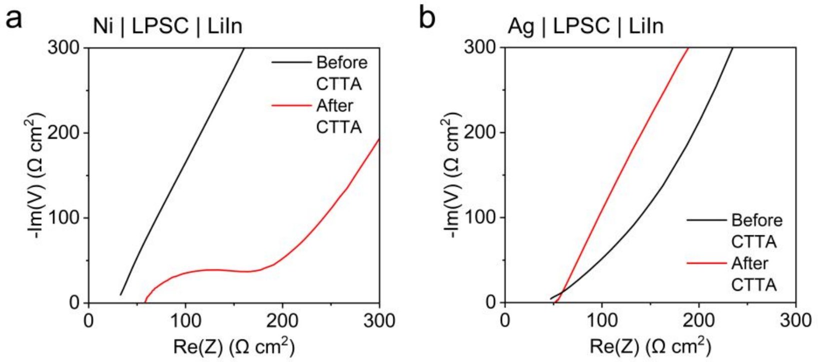

Figure S13. EIS analysis before and after 400 h of CTTA under 50 MPa stack pressure for (a) bare Ni and (b) 100 nm Ag-coated Ni electrodes.

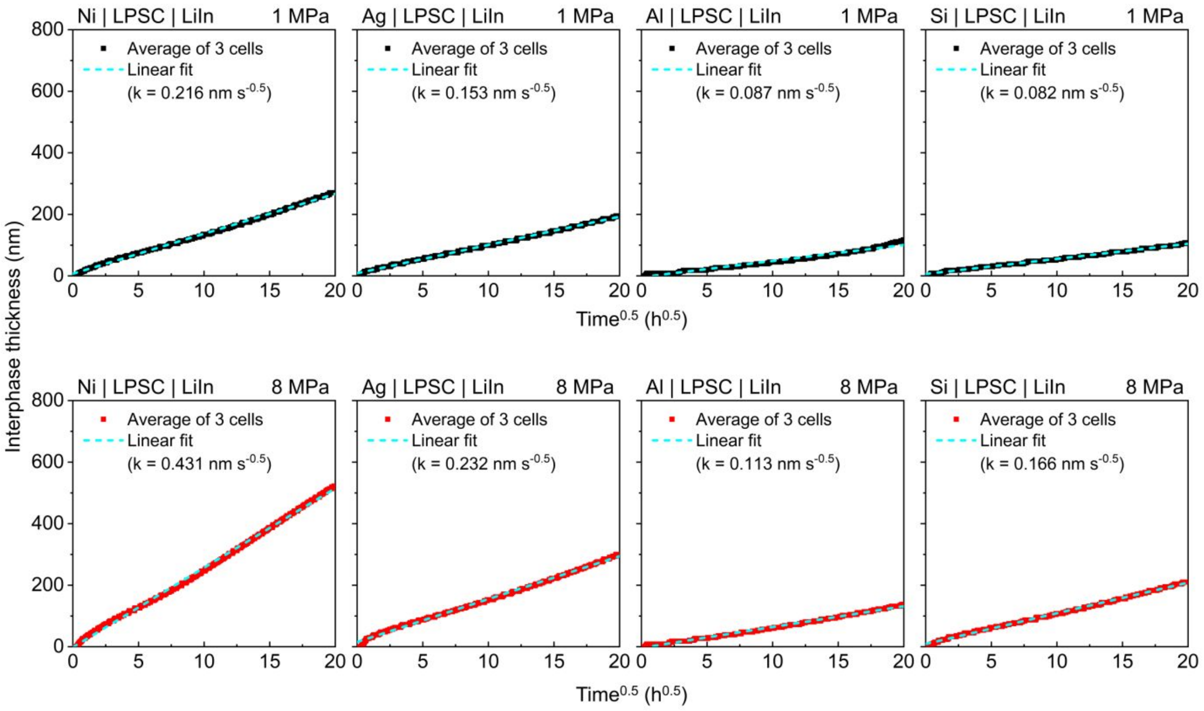

Figure S14. Calculated interphase thickness plotted as a function of square root of time ($h^{0.5}$) during 400 h of CTTA for bare Ni and 100 nm Ag-, Al-, Si-, and Ge-coated Ni tested under different stack pressures. Plots for 1 and 8 MPa stack pressures were linearly fitted (interphase thickness = $k \times t^{0.5}$) to extract parabolic constant (k) values.

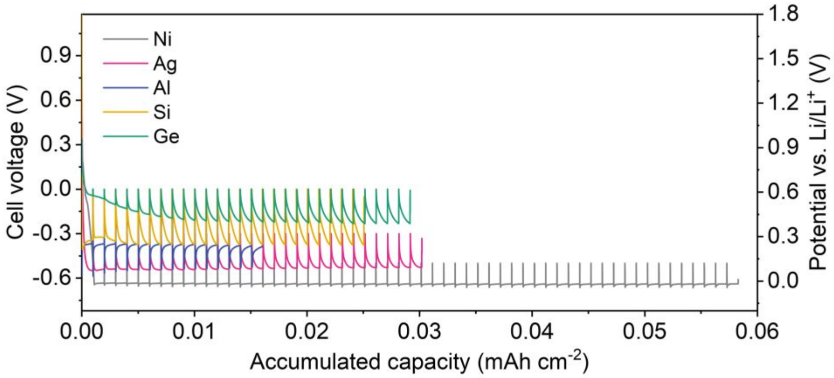

Figure S15. Electrode potentials of bare Ni, Ag-, Al-, Si-, and Ge-coated Ni electrodes plotted as a function of accumulated capacity (mAh cm^{-2}) during continuous titration steps tested under a stack pressure of 8 MPa.

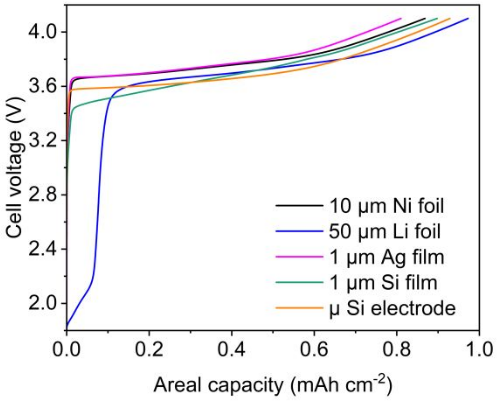

Figure S16. First charging curves of SSB full cells with different negative electrodes: bare Ni foil, 50 µm Li foil, 1 µm Ag-coated Ni, 1 µm Si-coated Ni, and slurry-cast Si microparticles. The full cells were charged under a stack pressure of 5 MPa at a current density of 0.25 mA cm^{-2}. For all cells, the cathode loading was 1.0 mAh cm^{-2}. $LiNb_{0.5}Ta_{0.5}O_3$ (LNTO)-coated $LiNi_{0.6}Mn_{0.2}Co_{0.2}O_2$ (NMC622) cathodes were used for all experiments.

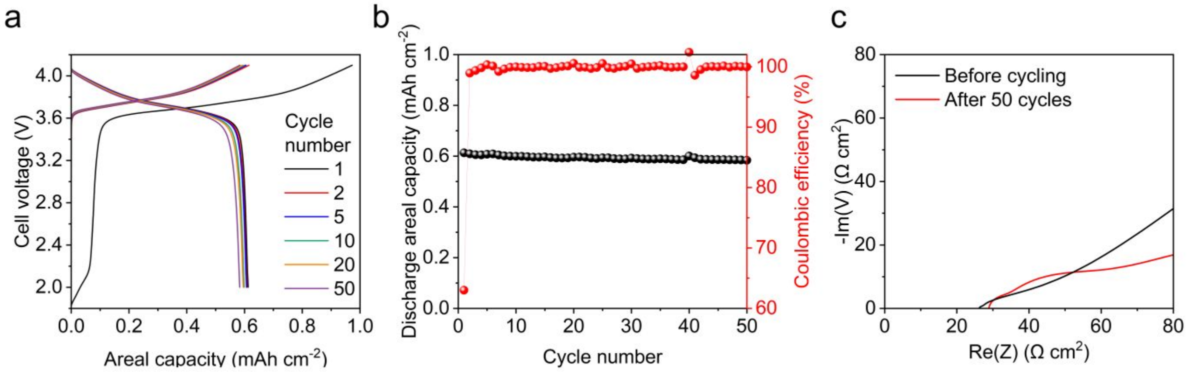

Figure S17. Galvanostatic cycling of a full cell with 50 µm Li foil as the negative electrode. (a) Galvanostatic voltage curves from the cycling test. (b) Discharge capacity during galvanostatic cycling. (c) EIS spectra of the cell before and after 50 cycles. This cell was cycled under a stack pressure of 5 MPa at a current density of 0.25 mA cm^{-2}. The cathode loading was 1.0 mAh cm^{-2}.

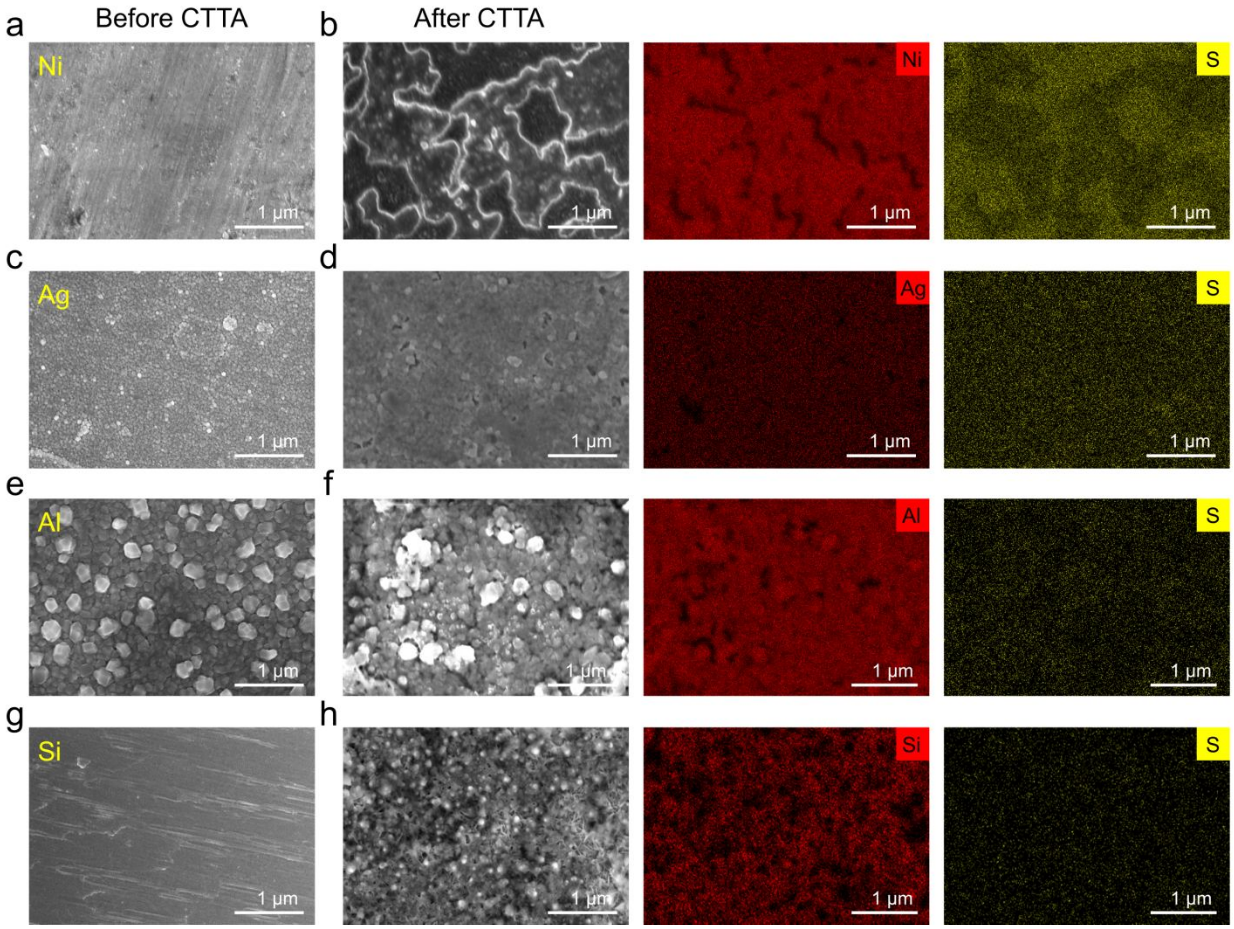

Figure S18. SEM images and corresponding EDS maps of electrodes before and after 400 h of CTTA under 50 MPa stack pressure. (a, b) SEM images and EDS maps of Ni and S elements for the bare Ni current collector (a) before and (b) after CTTA. (c, d) SEM images and EDS maps of Ag and S for the Ag-coated Ni electrode (c) before and (d) after CTTA. (e, f) SEM images and EDS maps of Al and S for the Al-coated Ni electrode (e) before and (f) after CTTA. (g, h) SEM images and EDS maps of Si and S for the Si-coated Ni electrode (g) before and (h) after CTTA.

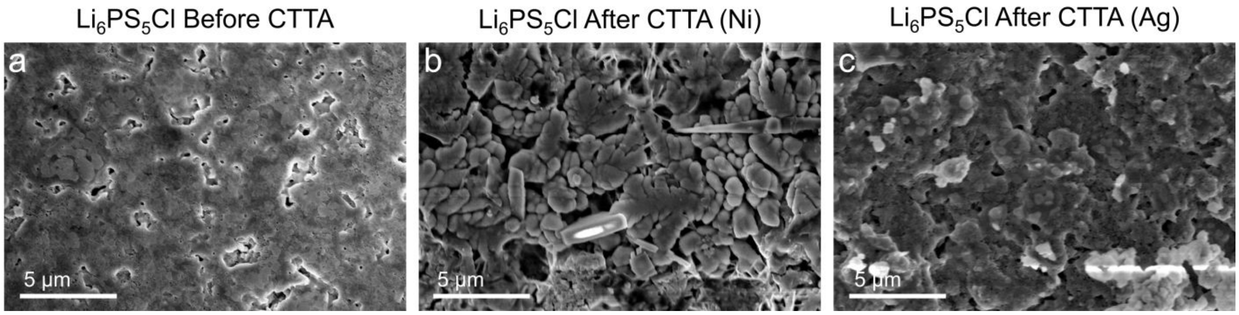

Figure S19. SEM images of (a) Li_6PS_5Cl pellet before CTTA and (b, c) Li_6PS_5Cl after 400 h of CTTA under stack pressures of 50 MPa in SSB cells with (b) a bare Ni electrode and (c) an Ag-coated Ni electrode.

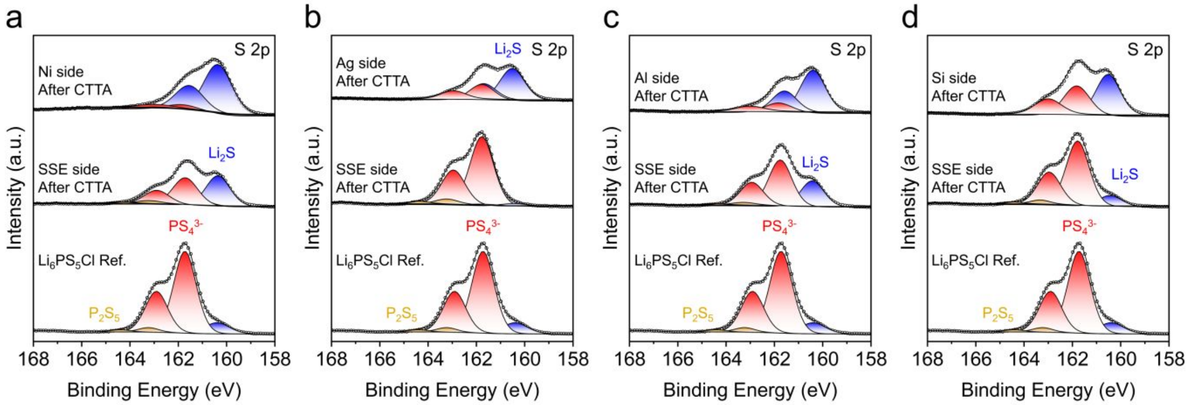

Figure S20. XPS S 2p spectra of Li_6PS_5Cl reference material, as well as spectra after 400 h of CTTA from both the electrode side and the SSE side of different electrodes: (a) bare Ni, (b) Ag-coated Ni, (c) Al-coated Ni, and (d) Si-coated Ni. 50 MPa stack pressure was used.

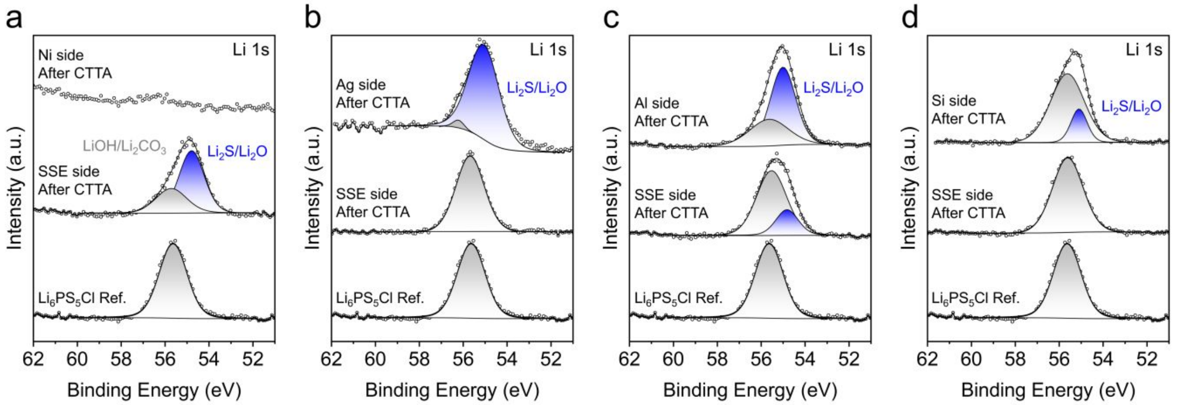

Figure S21. XPS Li 1s spectra of Li_6PS_5Cl reference material, as well as spectra after 400 h of CTTA from both the electrode side and the SSE side of different electrodes: (a) bare Ni, (b) Ag-coated Ni, (c) Al-coated Ni, and (d) Si-coated Ni. 50 MPa stack pressure was used.

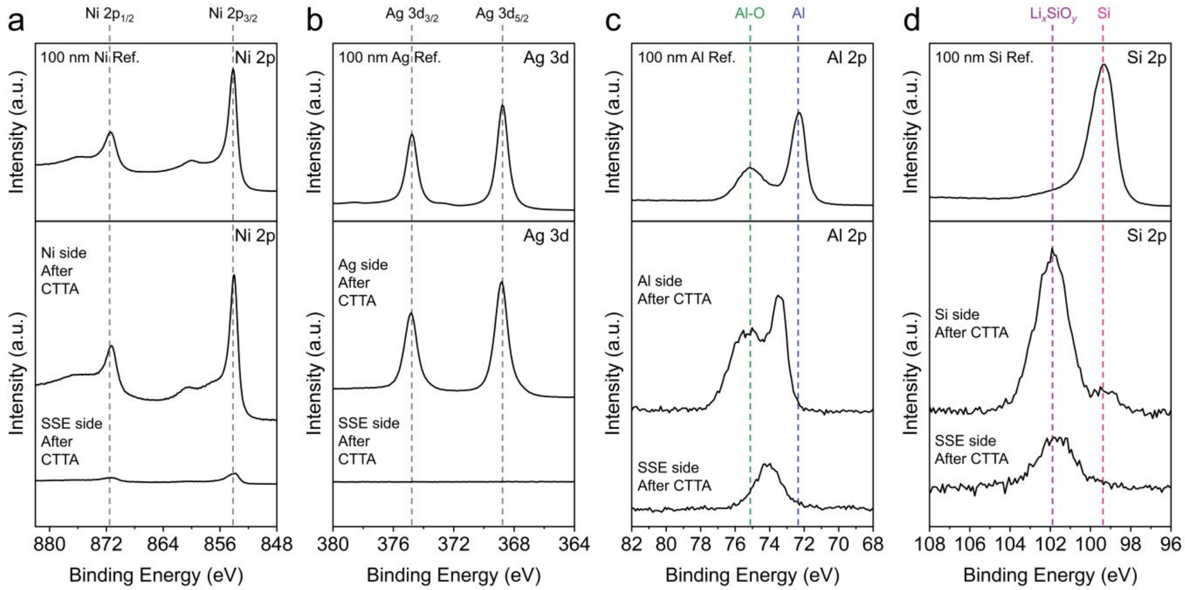

Figure S22. XPS spectra of pristine Ni various pristine deposited thin films (Ag, Al, and Si), as well as the electrode materials after 400 h of CTTA measurements, interrogating both the SSE side and the electrode side. (a) Ni 2p spectra from a cell with a bare Ni electrode, (b) Ag 3d spectra from a cell with an Ag-coated Ni electrode, (c) Al 2p spectra from a cell with an Al-coated Ni electrode, and (d) Si 2p spectra from a cell with a Si-coated Ni electrode. XPS measurements were conducted on samples from SSB stacks subjected to 400 h of CTTA under 50 MPa stack pressure.
